# Angular grain fragmentation with DEM modeling: application to fault gouge shearing

**DOI:** 10.1007/s10035-025-01578-9

**Published:** 2025-10-21

**Authors:** Nathalie Casas, Guilhem Mollon, Marco Maria Scuderi

**Affiliations:** 1 Dipartimento di Scienze della Terra, La Sapienza Università di Roma, Rome, Italy; 2 LaMCoS, CNRS UMR5259, INSA Lyon, Lyon, France

**Keywords:** Fragmentation, DEM, Sheared granular gouge, Angular grains

## Abstract

Understanding grain fragmentation in fault gouge is essential for capturing the mechanical behavior and evolution of fault zones under shear. In this study, we present a 2D Discrete Element Method (2D-DEM) framework that simulates comminution using angular, breakable grains, overcoming limitations of traditional models based on spherical particles. Our approach incorporates realistic fracture mechanics and grain geometries to better represent microstructural evolution during shearing. A series of numerical experiments, including Brazilian, oedometric, and shear tests, were conducted to calibrate the model and examine the roles of grain strength, friction, and Young’s modulus. The simulations reproduce key numerical observations such as strain localization, force chain evolution, and grain rounding through chipping mechanisms. Results show that the model captures the onset and progression of fragmentation, as well as its impact on fault strength and mechanical stability. A comparison with a dedicated laboratory experiment is provided. This work provides a robust numerical tool for studying fault gouge behavior and lays the foundation for future studies exploring the influence of initial grain size and material properties on fault mechanics.

**Graphical Abstract:**

**Figure:**
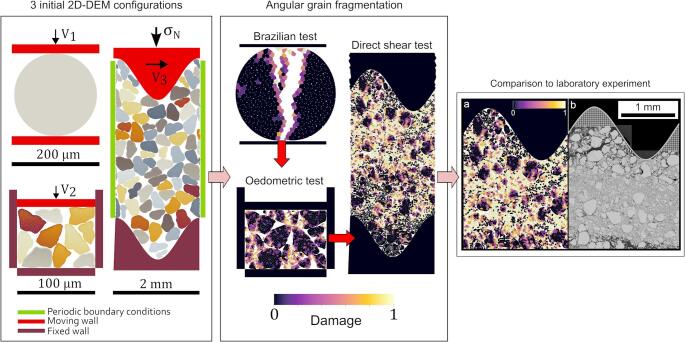
A robust numerical tool with fragmentation for studying fault gouge behavior

**Supplementary Information:**

The online version contains supplementary material available at 10.1007/s10035-025-01578-9.

## Introduction

Fault gouge, a granular material generated by wear between two host rocks along a fault, undergoes continuous grain fragmentation due to shearing under confining stresses of tens to hundreds of MPa [1–3]. Laboratory experiments on simulated fault gouge have demonstrated that grain comminution plays a key role in controlling fault strength, stability, and slip localization [4–10]. However, capturing these processes numerically remains challenging due to the complexity of fracture dynamics and the continuous evolution of microstructure within the gouge.

Grain fragmentation (e.g., comminution) is a fundamental mechanical process characterized by the progressive breaking of grains under stress. Mechanically, fragmentation is driven by the interplay of tensile, compressive, and shear stresses that exceed the material’s strength, leading to grain fracture and the formation of smaller fragments [11]. This process is crucial in understanding deformation mechanisms in highly loaded granular materials [12, 13], providing insights into the propagation of fractures within grains and their impact on fault frictional behavior under localized stress conditions. Unlike typical granular physics or soil mechanics experiments, the stresses encountered in fault gouge range from 0.1 to 300 MPa. Both in the field and in the laboratory [14], and they are sometimes comparable to the cohesive strength of the constituent rock [15].

Numerical modeling, particularly the Discrete Element Method (DEM), has been widely employed to simulate frictional and contact properties within fault cores [16–23]. One common approach is to simulate different grain sizes to study their effects on shear deformation [24, 25]. However, discrepancies in shear localization between simulations and laboratory results often arise due to the absence of dynamic fragmentation mechanisms, which play a crucial role in altering porosity and force chain distributions over time. Some models attempt to address this by incorporating bi-disperse or heterogeneous grain mixtures [24, 26], but these approaches fail to capture the continuous evolution of particle size, potentially distorting shear localization processes. Incorporating particle fragmentation into DEM has proven to be essential for accurately modeling grain breakage [27–30] and specifically during fault gouge shearing [31–33]. These models demonstrate the transition from early, localized fracturing to more distributed breakage events and highlight the interplay between intra- and inter-granular fracturing. Wang and collaborators [33] further demonstrated that grain breakage during seismic cycles can be triggered either by highly stressed force chains during stick phases or by grain rearrangement following slip events. However, most of these numerical models use spherical particles, limiting the accurate representation of angularity, realistic contact mechanics, and geometric heterogeneity observed in natural grains.

This paper presents a 2D DEM-based tool designed to simulate grain fragmentation with angular particles, offering a significant improvement over traditional models that rely on simplified grain geometries. Our model explicitly incorporates the irregular shapes of fault gouge grains, which play a critical role in fracture propagation, force transmission, and bulk material behavior. It extends the classical DEM framework with a novel proposition to simulate grain breakage, accounting for more realistic fragment formation. The primary objective is to numerically replicate grain fragmentation during fault gouge shearing, facilitating direct comparison with laboratory experiments. To achieve this, we use a sequence of numerical models: a Brazilian test to simulate single-grain fragmentation, an oedometric model to assess multi-grain breakage under compression, and a shearing fault gouge model to capture progressive particle breakage, grain rearrangement, and potential strain localization. We validate the shearing fault gouge model by comparing its results with those from a dedicated laboratory experiment. This refined approach allows for the choice of the appropriate set of numerical parameters while providing deeper insights into the micro-mechanical processes driving fault behavior.

## General method

###  Numerical modeling with angular grains

This study employs the MELODY2D code [34, 35] to perform 2D Discrete Element Modeling (DEM) of granular materials, providing a robust framework for simulating the motion and interactions of individual particles. The code is capable of handling a large number of rigid or deformable bodies with arbitrary geometries in a unified manner. Each particle follows its trajectory governed by Newton’s laws of motion, allowing the model to capture the granular system’s dynamic behavior. Newtonian dynamics of rigid bodies are solved in time using classical DEM contact interactions, ensuring consistent and accurate results. MELODY2D is written in C + + and parallelized in an OpenMP framework.

Angular particles are used in the simulations, emphasizing the significance of particle shape in determining the effective frictional properties of granular materials, as highlighted in previous studies [25, 36–39]. Non-circular geometries are critical for realistic representations of fault gouge mechanics, as they significantly influence the mechanical response under stress.

### Grain fragmentation and contact algorithm

**Fig. 1:**
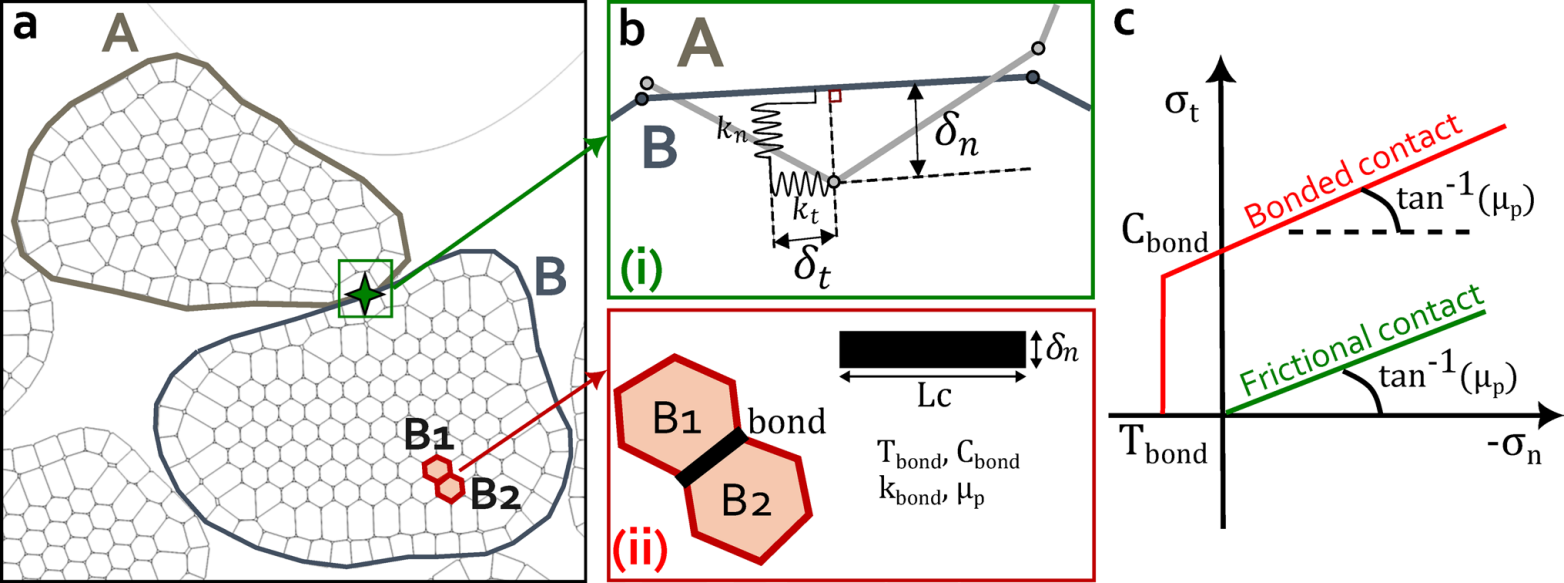
**a** New discretization of angular grain into subgrains.** b** Sketch of grain contact interaction for the BMC contact law from MELODY: (i) broken (unbonded) contact and (ii) intact (bonded) contact.** c** Contact model for bonded and unbonded grains or BMC contact law

To enable grain fragmentation, angular particles are discretized into smaller polygonal subgrains (Fig. 1a). To build synthetic angular samples, we first employ the Fourier-Voronoi technique [40], adapting the coefficients of the Fourier spectrum to achieve a good visual match with typical gouge grains. Then, to create the subgrains, we set on each grain a cloud of points, along a hexagonal lattice disturbed by a user-defined random noise, and these points are used as seeds for a bounded Voronoi tessellation on the grain domain. Each Voronoi cell is then used as a “subgrain”. This subgrain discretization is a novel contribution to MELODY2D and allows for the simulation of varying material properties, as subgrains can detach and move independently once the applied stress surpasses the cohesive forces binding them. The size and geometric regularity of the subgrains are user-defined, offering flexibility for diverse simulation scenarios. Indeed, in this work, the polygonal shape of subgrains is not perfectly regular to enable a certain randomness within the distribution of particles. Particle contours are modeled using piecewise linear boundaries, and interpenetrations are prevented through a two-pass node-to-segment contact algorithm, described in detail in [34, 41].

In this study, grain contact interactions within the model are governed by physics-based contact laws. Local contact parameters are chosen in such a way that the upper scale behaves in the desired physical way, but may not necessarily have a physical interpretation. We use the Bonded Mohr-Coulomb (BMC) contact law to model grain breakage, previously implemented and detailed by Casas et al. [41], to simulate cementation within a fault gouge. This contact law is conceptually similar to the bonded-particle model (BPM) proposed by Potyondy and Cundall [42], where the contact bond between two particles operates in two statuses (intact or broken), controlling fragmentation. A subgrain detachment (e.g., grain fragmentation) occurs when the stress exceeds the cohesion threshold for all the contacting sides of a subgrain, implying the suppression of the initial bond [41]. The two statuses of a given bond, (i) broken or (ii) intact, are described below:

(i) Broken (or unbonded free) contact

This type of contact status model standard and non-cohesive interactions, where forces are determined by classical DEM parameters such as contact friction ($\:{\mu\:}_{p}$), normal and tangential contact stiffnesses ($\:{k}_{n}$ and $\:{k}_{t}$), and numerical damping, ensuring realistic mechanical behavior (Fig. 1b i). These contacts can be observed in three scenarios: between large grains (e.g., Fig. 1a, grains A and B), between subgrains/large grains and lateral walls, or between two subgrains after particle fragmentation (e.g., broken bond). Between two unbonded subgrains (broken contact), the normal ($\:{\sigma\:}_{n}$) and tangential ($\:{\sigma\:}_{t}$) contact stresses are computed based on the respective normal and tangential overlapping gaps ($\:{\delta\:}_{n}$ and $\:{\delta\:}_{t}$), computed geometrically within the code for each time step. Under typical applied normal stresses and selected contact stiffnesses, grain interpenetrations remain below 1% of the particle size, maintaining the physical plausibility of deformation mechanics.

These relationships are represented schematically in Fig. 1b(i) and the following equations:

no contact

1 $$\:{if\:({\delta\:}_{n}>0)\:\to\:{\sigma\:}_{n}}_{\:}={\sigma\:}_{t}=0$$

contact

2 $$\:{if\:({\delta\:}_{n}<0)\:\to\:{\sigma\:}_{n}}_{\:}={k}_{n\:}{\delta\:}_{n}\:\:\:\:\&\:\:{\:\sigma\:}_{t}=min({k}_{t\:}{\delta\:}_{t},{\mu\:}_{p\:}{\sigma\:}_{n})$$

The contact friction $\:{\mu\:}_{p}$ and the normal and tangential contact stiffnesses ($\:{k}_{n\:}$and $\:{k}_{t}$) are user-defined and chosen to correspond to the material or rock we want to model. They stay constant during the entire simulation. The tangential and tensile cohesive stresses for a broken contact ($\:{C}_{free}\:\&\:{T}_{free}$) are set equal to zero since once the contact is broken and will not be able to become cohesive anymore within this simulation. A normalized damping of 0.3 is applied to dissipate energy at each contact.

(ii) Intact (or bonded) contact

This type of contact status represents cohesive interactions between grains. It is observed initially between all subgrains within one large grain (Fig. 1a, grain A). In this type of contact, the normal gap computation depends on the properties of the bond chosen by the user: $\:{\delta\:}_{n}$< ($\:{\delta\:}_{detection}=\:{C}_{bond\:}/{k}_{bond})$, where $\:{k}_{bond}$ is the bond contact stiffness and $\:{C}_{bond\:}$ is the cohesive stress, representing the cohesion threshold to exceed to break the bond (Fig. 1b (ii)).

3 $$ \:\sigma \:_{n} = k_{{n\:}} \delta \:_{n} \:\:\:\:\& \:\:\:\sigma \:_{t} = k_{{t\:}} \delta \:_{t} \:\: $$

If $\:{\sigma\:}_{n}$ (respectively $\:{|\sigma\:}_{t}|$), the normal (respectively, tangential) stress exceeds the prescribed value of $\:{T}_{bond\:}$ (respectively $\:{C}_{bond\:}$), the status of the contact is updated to ‘‘broken’’ (Fig. 1c). In the bonded contact, both tangential and tensile cohesive stresses ($\:{C}_{bond}\:\&\:\:{T}_{bond})\:$ are initially defined. We can either assign them identical or different values.

From these stresses, the associated contact forces (in the normal and tangential directions) are computed on each grain by considering that contact stresses act on a contact length $\:{L}_{c\:}$(equal to the sum of half-lengths of the segments around contact nodes in grain A, Fig. 1b):

4 $$ F_{n} = L_{{_{c} }} \sigma _{n}\:\:\&\:\:F_{t} = L_{c} \sigma _{t} $$

As for the broken contact, the contact friction $\:{\mu\:}_{p}$ and the normal and tangential contact stiffnesses ($\:{k}_{n\:}$and $\:{k}_{t}$) are user-defined parameters set equal for both broken and intact contacts. All the bonds within each large grain, in its intact status, give an elastic character to the grain before the bond breaks [43].

### Numerical models

**Fig. 2:**
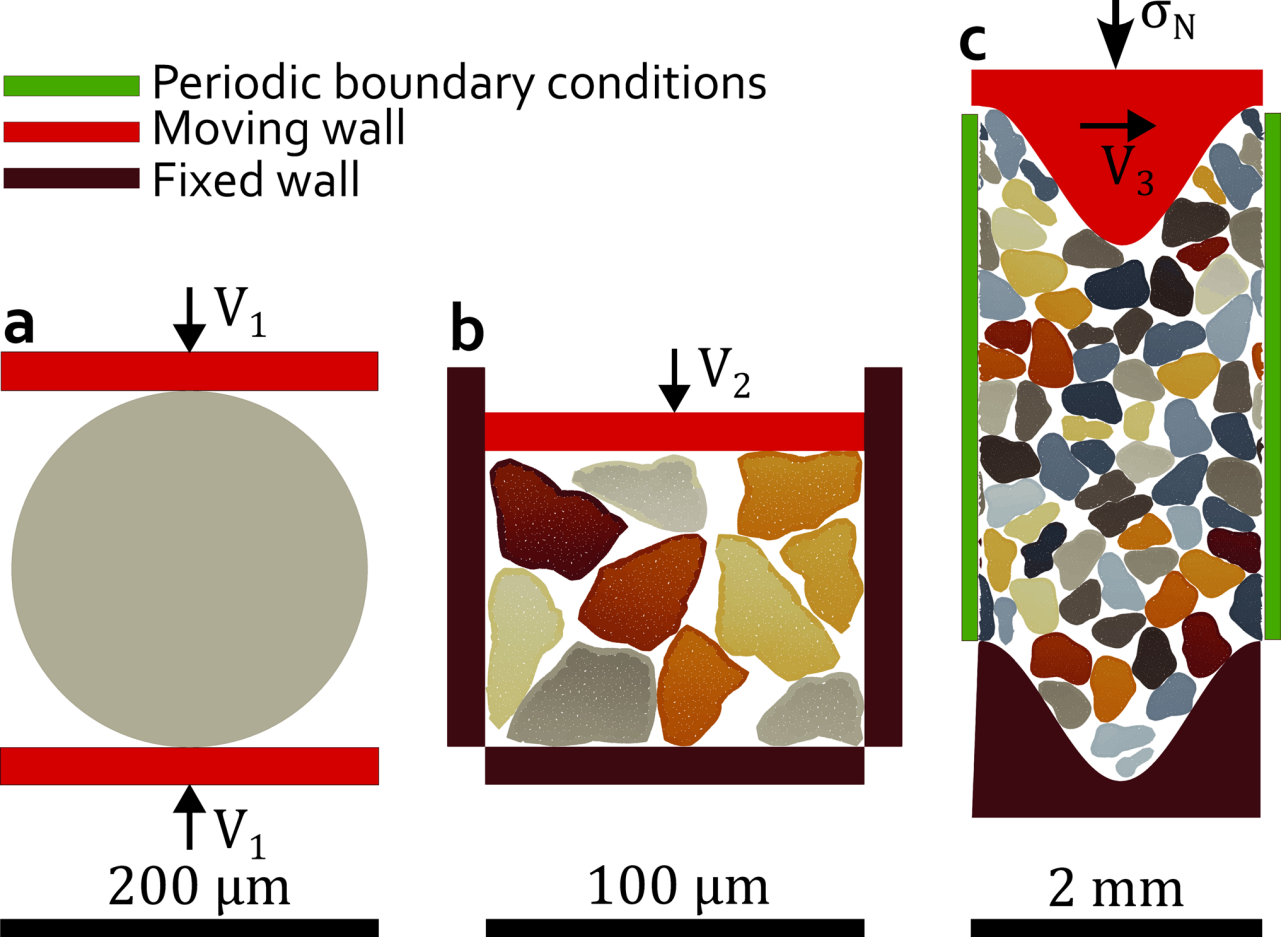
**a** Brazilian test model, with one grain.** b** Oedometric test with 10 grains.** c** Fault gouge model with 70 grains. For the vertical imposed boundary condition, the Brazilian test numerical model is velocity-driven, and the two walls are moving toward the grain at a constant velocity $\:{V}_{1}$. The top wall of the oedometric test is velocity-driven at a constant velocity $\:{V}_{2}$. A constant normal stress $\:{\sigma\:}_{n}$ and a constant shearing velocity $\:{V}_{3}\:$are applied to the top wall of the fault gouge model

To evaluate grain fragmentation and benchmark the model’s performance, a series of 2D numerical setups with increasing complexity are implemented. The first configuration, a Brazilian test, simulates a single circular grain under displacement-controlled diametral loading to assess fundamental fracture mechanics and sensitivity to parameter variations (Fig. 2a). Then, an oedometric test extends this approach to a system of 10 angular grains, allowing for the examination of fragmentation in multiple grains subjected to compaction and the influence of key mechanical parameters (Fig. 2b). Finally, the most complex setup, a fault gouge model inspired by the setup of laboratory experiments [44], consists of 70 angular grains undergoing both compaction and subsequent shear, enabling the study of emerging frictional response as the gouge’s internal state evolves under the (BMC) contact law (Fig. 2c). This progressive increase in complexity, from a single grain to multiple grains and then to a sheared fault gouge, allows us to systematically refine our numerical model. The final gouge model aims to capture the interplay between grain breakage, particle rearrangement, and strain localization, which are critical to fault mechanics.

To assess grain fragmentation within our model, we track the damage experienced by each grain during the simulation. Every time a bond breaks between two subgrains, the damage value assigned to each subgrain increases. Damage values range from 0 for grains with all initial bonds intact to 1 for grains where all bonds have been broken. The evolution of damage is then monitored throughout the simulation. At selected time steps, we calculate the average damage (the mean damage across all grains), providing insight into the overall fragmentation process.

## Single-grain fragmentation

### Numerical setup for a Brazilian test

This Brazilian test model provides an idealized representation of the loading conditions experienced by a single grain, serving as the foundation for the implementation of subsequent models with multiple grains. The model’s geometry is based on classical laboratory Brazilian tests [45–47]. The setup consists of a single disc-shaped grain placed between two rectangular forcing blocks (Fig. 2a). The disc has a diameter of D = 200 μm and is subdivided into $\:\sim400$ irregular polygonal subgrains (e.g., Fig. 1a), each with an average diameter of d = 10 μm (unless specified otherwise). During the simulation, the forcing blocks move vertically at a constant velocity *V*_1_ = 1 mm/s, applying compressive loading to the disc.

The BMC contact law governs grain contact interactions during this simulation (Sect. 2.2). The numerical parameters for the reference models are summarized in Table 1. It is to be mentioned that the parameters tested in this first model are numerical local parameters that are not necessarily expected to have any strong physical meaning by themselves. They are chosen in such a way that the upper scale (here, the grain mechanical response as explored in the next two models) behaves in the desired physical way. To investigate the local parameters influencing grain breakage, a series of sensitivity analyses were performed by systematically varying model properties:

- **Contact stiffnesses.** The normal and tangential contact stiffnesses $\:{k}_{n\:}$ and $\:{k}_{t\:}$—controlling contact interactions between particles—were adjusted to alter the equivalent Young’s modulus of the entire disc, following the methodology described by Casas et al. [25]. Simulations were conducted with values $\:{k}_{n\:}={k}_{t\:}$ equal to 5.6e15 Pa/m (reference case, Table 1), as well as 4.2e15, 2.8e15, 1.4e15 Pa/m.
- **Contact cohesive stresses.** The tangential and tensile cohesive stresses, $\:{C}_{bond}\:\&\:{T}_{bond}$​, are key parameters as they determine the cohesive forces that must be overcome to fracture the grain. They were systematically varied between 50 MPa and 500 MPa. For the reference model, $\:{C}_{bond}\:\&\:{T}_{bond}$​ are set equal to 300 MPa (Table 1).
- **Contact friction.** The contact friction coefficient $\:{\mu\:}_{p}$ quantifies the friction value at the interface of each grain contact. This is a local and user-defined (numerical parameter) friction value varied within a range of 0.3 to 0.7, applied to both intact and broken contacts, to assess their influence on grain failure and post-fracture interactions. For the reference model, $\:{\mu\:}_{p}$ is set equal to 0.5 (Table 1).
- **Grain confinement.** Additional simulations introduce lateral boundaries (confinement) to the model shown in Fig. 2a, applying lateral stress to simulate constrained grain behavior.
- **Size of the subgrains**. The influence of subgrain size d on fragmentation was analyzed to determine whether a maximum admissible subgrain size exists relative to the size of the larger grain. The size of subgrains is a key parameter since it could influence how the large grain breaks; it varied between 3 and 20 μm. For each grain size, a cohort of 6 to 20 simulations was performed to evaluate the repeatability of fragmentation patterns and mechanical responses.

Additional parameters of the contact law were also tested to check that they did not have any major effect on the grain behavior (Supporting Information S1). For each simulation, the equivalent stress (calculated as the axial force divided by the disc diameter) was plotted alongside axial strain (calculated as the relative shortening of the grain in the vertical direction) to evaluate the mechanical response of grains under different conditions. The evolution of damage was continuously tracked across all configurations, providing insight into how each parameter affects fracture initiation and propagation.

**Table 1:** Numerical parameters used for the Brazilian test model “reference case”, with the bonded Mohr-Coulomb (BMC) contact law

Parameters	Value	Parameters	Value
*k*_*n*_	5.6^e^15 Pa/m	ϒ	0.3
*k*_*t*_	5.6^e^15 Pa/m	*C*_*free*_	0 MPa
*k*_*bond*_	5.6^e^15 Pa/m	*T*_*free*_	0 MPa
*T*_*bond*_	300 MPa	$\:{\boldsymbol{\mu\:}}_{p}$	0.5
*C*_*bond*_	300 MPa		

### Results

**Fig. 3:**
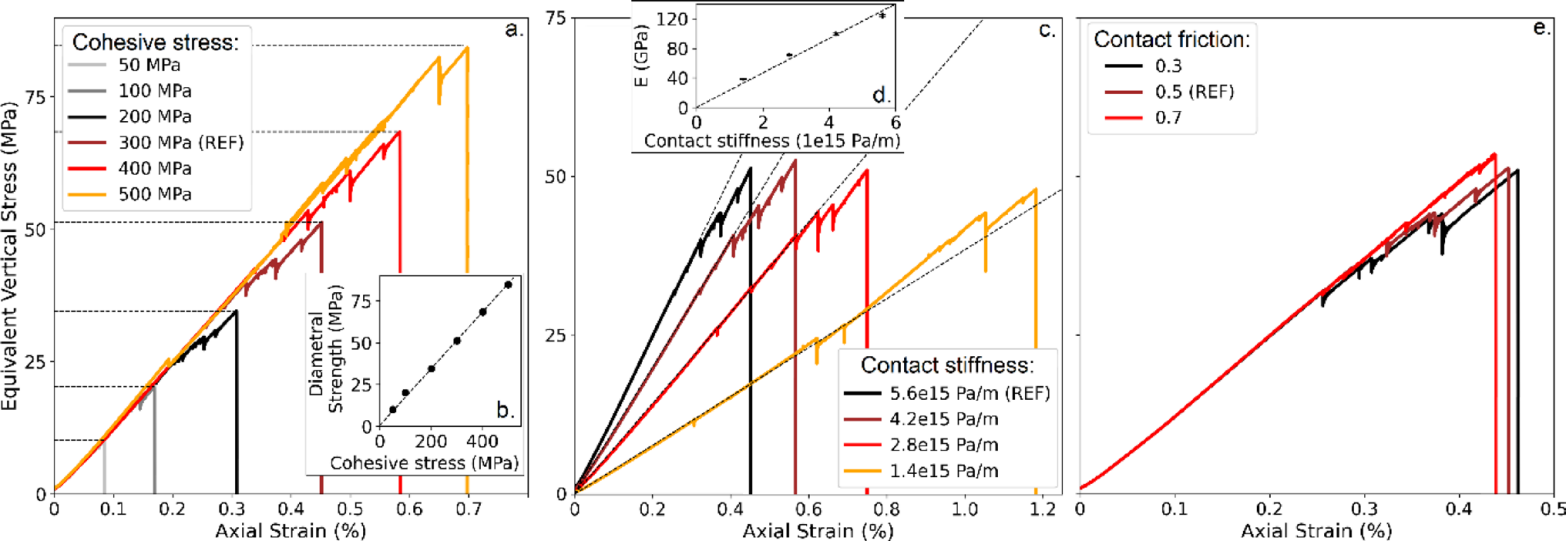
Influence of contact parameters on grain-scale mechanical behavior.** a** Stress-strain curves for various contact cohesive stresses;** b **Diametral strength (MPa) as a function of cohesive stress;** c** Stress-strain curves for various contact stiffnesses;** d** Young modulus as a function of contact stiffness;** e** Stress-strain curves for various contact friction

**Fig. 4:**
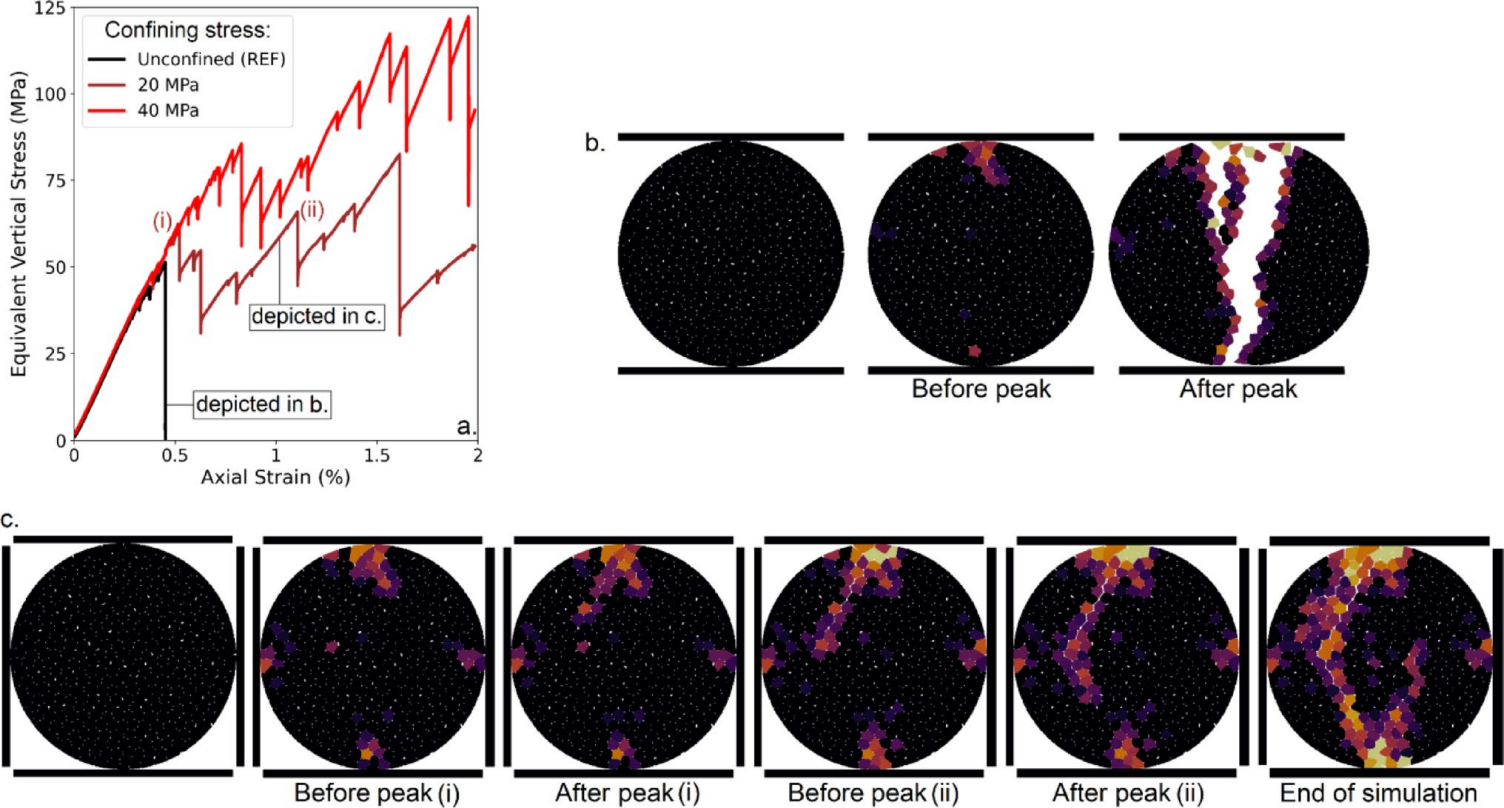
**a** Equivalent vertical stress (MPa) as a function of axial strain (%) under unconfined and laterally confined conditions. **b** Progressive breakage of a grain under unconfined conditions.** c** Progressive breakage of a grain under laterally confined conditions. Repeatability experiments can be found in Figure S2 (Supporting Information S2)

**Fig. 5:**
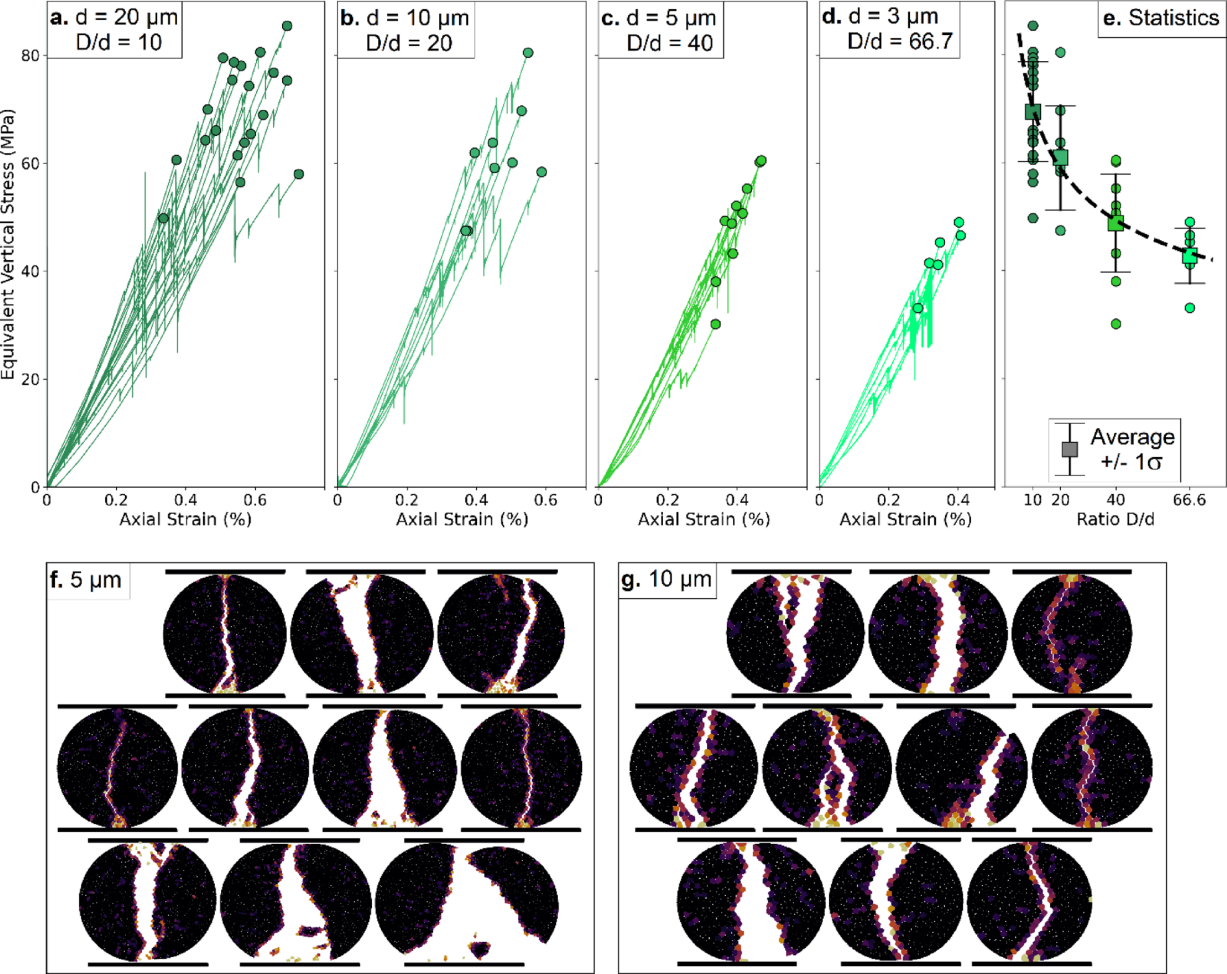
Equivalent vertical stress (MPa) as a function of axial strain (%), for different subgrain sizes:** a** 20 μm subgrains,** b**10 μm subgrains,** c** 5 μm subgrains, and** d** 3 μm subgrains.** e** Statistics of diametral strength across 6–20 simulations for each subgrain size as a function of the ratio D/d, and best fit on Weibull statistics (*m = 7.8*).** f** Final broken grain configurations for all 10 simulations with 5 μm subgrains.** g** Final broken grain configurations for all 10 simulations with 10 μm subgrains. In a., b., c., and d., post-failure portions of the curves were omitted for clarity

The results of sensitivity analyses with the Brazilian test model are presented in Figs. 3, 4 and 5, where the equivalent stress evolution (computed as the axial force divided by the disc diameter) and axial strain are plotted for each simulation:

#### Contact cohesive stresses

To simplify this initial test, the contact cohesive stresses, $\:{C}_{bond}\:\&\:{T}_{bond}$, are set equal. As expected, the value of cohesive stresses directly influences the stress required for failure (also called “diametral strength” hereafter): higher cohesive stresses result in increased vertical stress and greater axial strain before grain breakage occurs (Fig. 3a). The evolution of vertical stress with strain shows an approximately linear relationship before failure, but does not affect the failure mode, which remains characterized by sudden brittle fracture under these loading conditions (Fig. 3a). In the range we explored, we obtained a linear relationship between cohesive stress and diametral strength, with a ratio close to 0.17 (Fig. 3b).

Cohesive stresses are then adjusted with relative ratios of 0.5, 1 and 1.5 to examine their differential effects on grain cohesion. Their variation appears to have a minimal impact on single-grain breakage (Figure S1b, Supporting Information S1). This suggests that while the value of the cohesive stress significantly influences the stress threshold for failure, variations in the relative ratio of tangential to tensile cohesion have little effect on the overall fracture behavior of an isolated grain. These results align with the nature of the numerical configuration (Brazilian test), where most of the bonds fail in tension and not in shear.

#### Contact stiffnesses

The contact stiffnesses, $\:{k}_{n\:}$ and $\:{k}_{t},\:$were adjusted to modify the equivalent Young’s modulus of the disc and assess its impact on grain breakage. Results indicate that the contact stiffness controls the emerging elasticity of the grain before its breakage. It also influences the axial strain at which the first failure event occurs (Fig. 3c). Specifically, lower contact stiffness results in higher axial strain needed for grain breakage. However, the equivalent vertical stress at failure remains essentially unaffected (Fig. 3c). It has to be remembered that these contact stiffnesses are local (and non-physical) parameters designed to calibrate the equivalent grain models. So, in this case, by modifying contact stiffnesses, we can control the elasticity of the grain without changing its strength. From the linear part of each stress-strain curve, an equivalent Young modulus of the grain can be extracted. This is performed by numerically integrating the classical expressions of the strain field in a diametrically loaded elastic disc [48, 49] and running a back-analysis. In the explored range, we obtain a linear relationship between contact stiffness and Young modulus (Fig. 3d).

In contrast, the tensile bond stiffness ($\:{k}_{bond\:}$) appears to have minimal influence on grain breakage, as demonstrated in Figure S1a (Supporting Information S1). This suggests that while contact stiffness directly affects the axial strain required for failure, tensile bond stiffness plays a negligible role in determining the breakage behavior of the grain.

#### Contact friction

The impact of contact friction $\:{\mu\:}_{p}$ (local friction set numerically at each contact) on the internal breakage of a single grain under external loading seems undocumented. To assess this, we varied the contact friction from 0.3 to 0.7 and analyzed its effect on grain fracture. As illustrated in Fig. 3e, contact friction showed no significant influence on the breakage process, and the rupture remained brittle. This parameter is a necessary ingredient to the contact law, as it is of primary importance for intergranular contacts (e.g., contacts between different grains, before or after breakage). However, these findings suggest that, under the tested conditions, contact friction does not interfere with breakage in the Brazilian configuration (because grains essentially break in tension in that case), and that it can be calibrated independently for other purposes (e.g., intergranular friction).

#### Confinement

The failure behavior of an unconfined disc is compared to that of a disc subjected to lateral pressure applied via two additional walls. This last setup more closely replicates the conditions experienced by grains in an oedometric test while maintaining a simplified configuration. As expected, confinement significantly alters the failure mechanism (Fig. 4a). Under confinement, the grain can withstand higher stresses and sustain deformation for a considerably longer axial strain before failure. In the images (Fig. 4b and c), the evolution of grain breakage is compared between the confined and unconfined cases. Notably, confinement delays the onset of the first rupture, but it also enables subsequent failure events, which are absent in the unconfined scenario. A more detailed analysis of progressive grain failure is provided in the Supporting Information (Video S1). To assess the repeatability of these confined tests, we repeated the experiment on ten grains under identical conditions (20 MPa lateral confinement). Results show consistent trends, although individual grain responses vary in post-peak strength response, reflecting the influence of subgrain shapes and internal structure on failure behavior (Figure S2, Supporting Information S2).

#### Impact of subgrain size and repeatability

The influence of subgrain size $\:d$ on fragmentation was analyzed with respect to the size of the large grain $\:D$, kept constant at 200 μm. For each subgrain size, a cohort of 6 to 20 simulations (depending on the computational cost and the expected response dispersion) was conducted to evaluate the variability of strength, allowing us to assess the repeatability of grain breakage. Figure 5 illustrates the evolution of vertical stress as a function of axial strain for four subgrain sizes: 3, 5, 10, and 20 μm. Raw results (Fig. 5a–d) indicate, as a general trend, that the strength of a grain submitted to diametral compression decreases when the size of the subgrains is smaller. The data are compiled in Fig. 5e, where the individual strength of each tested grain, as well as the average value and standard deviation for each subgrain size, are plotted as a function of the ratio D/d. This ratio describes the typical number of subgrains contained in the diameter of the large grain, but it can also be seen as the ratio between the grain size and the typical mechanical defect. Results show that the mean strength decreases with D/d in a regular manner, which is consistent with Weibull statistics [50]:

5 $$ \:\sigma \:_{f} \propto \:\left( {\frac{D}{d}} \right)^{{ - \frac{2}{m}}} \:\: $$

The constant ‘2’ comes from the dimensionality of the system. The best fit in Fig. 5e provides a Weibull modulus $\:m=7.8$, which is in the typical expected range of 6–30 [50]. It is worth mentioning that the usual interpretation of Weibull statistics expressed in Eq. (5) is based on a varying specimen size while the size distribution of the defects is kept constant, but the underlying physics are dimensionless: what matters is the ratio between the respective sizes of defects and specimen, materialized in the present case by the ratio D/d. The assumptions of the model and the chosen contact law therefore provide results in agreement with the literature, if the parameter $\:d$ is seen as a physical parameter (scale of the defects in the rock grains) instead of merely a discretization parameter to be chosen as small as possible. Figure 5f and g present the fractured grains from each of the 10 simulations for the 5 and 10 μm and subgrains. These results highlight that, despite similar equivalent vertical stress and axial strain values, the rupture pattern can vary considerably depending on the initial configuration of subgrains, which can slightly vary in shape (e.g., Sect. 3.1).

### Discussion

The numerical model for single-grain crushing presented in this section effectively captures grain breakage behavior and can be extended to multi-grain simulations within a DEM framework. In a Brazilian test configuration (diametral compression), the mechanical response of a grain primarily depends on two key local parameters: contact stiffness, which influences the bulk Young’s modulus without affecting grain strength, and contact cohesion between subgrains, which solely determines the ultimate load a grain can withstand before crushing. This distinction enables precise control of the bulk mechanical response by decoupling deformation from strength. Other micromechanical parameters, such as the contact friction of the rock material or the ratio between tensile and tangential strength, have only a minor impact on the overall response (Fig. 3 and S1b).

However, the presence of lateral confinement—introduced through localized contacts with lateral walls that simulate neighboring grains in a loaded sample—significantly affects grain breakage behavior. While conventional diametral loading results in a nearly linear-brittle response (Fig. 3), the presence of confinement alters this response substantially (Fig. 4). Confinement increases the load a grain can withstand, modifies the crack propagation path, and delays final breakage by introducing multiple intermediate stress drops (Fig. 4a). This type of configuration is expected to be more common for the fault gouge scenario than the conventional diametral loading in a granular sample, as each grain has a large number of contacting neighbors. At the scale of the whole sample, this effect likely contributes to increased ductility compared to the sudden failure in a purely diametral loading.

Tests performed using different levels of grain discretization into subgrains show that larger subgrains result in stronger grains and greater variability in vertical stress and axial strain between simulations. In contrast, grains composed of smaller subgrains tend to be weaker on average but exhibit more consistent mechanical responses. This observation is in qualitative (and even quantitative, Fig. 5e) agreement with common knowledge and observation that smaller specimens of a given rock are stronger than large ones, owing to the presence of more defects in the loaded domain. In our simulations, this manifests as a dependence of grain strength on the ratio between grain and subgrain size, e.g., on the number of potential failure planes within the large grain. As shown in Fig. 5e, this relationship follows Weibull statistics [50], which enable extrapolation of grain strength across different subgrain sizes. Accordingly, subgrain size should be carefully considered when designing numerical samples. Alternatively, it is possible to remove this size-dependence by taking advantage of the linear fit of Fig. 3b (diametral strength equals ~ 0.17 times the bond strength) and of Eq. 5 in order to assign manually to each grain the appropriate bond cohesive strength depending on its size.

The variability in strength observed from one grain to another reflects natural heterogeneity (Fig. 5f, g and S2) and exists for all values of $\:D/d$ (although it is more pronounced for coarse discretizations). This suggests that a moderate discretization, e.g., a grain-to-subgrain size ratio of ~ 10, is sufficient for scaling to larger systems without excessive computational cost. Beyond subgrain size, the structural disorder introduced during Voronoi discretization also plays a role in shaping mechanical behavior. Local geometric irregularities can influence fracture initiation, propagation paths, and sample-to-sample variability. While we did not explicitly vary this factor in the present study, the ratio of cohesive strength to diametral strength is likely sensitive to this internal disorder, and its influence on micromechanical responses warrants further investigation in future work.

The parameters tested in this initial model are numerical and local, without inherent physical significance. Instead, within the next models, we will use the Diametral Strength (instead of contact cohesive stresses) and the grain Young modulus (instead of contact stiffnesses), which will be tested to ensure that the larger-scale behavior models align with the expected physical response.

## Multiple-grain fragmentation

### Numerical setup for oedometric tests

The second type of 2D simulation in this study aims at replicating laboratory oedometric tests (also known as one-dimensional compressive tests). These experiments are widely used in soil mechanics and geotechnical engineering to investigate the compressibility and consolidation behavior of soils or granular materials under applied vertical stress [51, 52]. These simulations aim at determining the axial load required for grain breakage and exploring the role of Diametral strength ($\:Ds$) and grain Young modulus ($\:E$).

The numerical setup consists of 10 angular grains confined within a 100 × 100 μm^2^ square box, similar to a Representative Element Area of a fault gouge sample (Fig. 2b). The grains have equivalent diameters of about 25 μm and are subdivided into irregular polygonal subgrains with an average diameter of 2 μm (i.e., at most one-tenth of the average grain size) with around 160 subgrains per grain on average. The simulation applies compressive loading by moving the top block downward at a constant velocity *V*_2_ = 0.05 m/s. We checked that this velocity is sufficiently low to avoid any inertial effect in the system.

The BMC contact law governs the interactions between grains during the compaction phase, and the reference model’s numerical parameters are summarized in Table 2. To analyze the parameters influencing multiple grain breakage, a series of sensitivity analyses was conducted, guided by insights from the Brazilian test model results. The parameters investigated include:

- **Young modulus of a grain**: In the single-grain model, contact stiffness controlled the equivalent Young’s modulus $\:E$ of each grain and had a significant impact on the axial strain required for a single-grain breakage (Fig. 3b). $\:E$ was tested within the range of 4, 9.8, 38.1, and 89.8 GPa (corresponding to normal and tangential contact stiffness values, $\:{k}_{n}=\:{k}_{t}$, within the range 8e10¹⁴ to 2e10¹⁶ Pa/m), to evaluate its role in multi-grain fragmentation. Young’s modulus also affects the numerical contact overlap, a local parameter investigated in Sect. 3. These local moduli should not be confused with the bulk Young’s modulus, which is derived from the overall deformation of the entire sample.
- **Diametral strength**: In the single-grain model, increasing internal grain cohesion resulted in a linear increase in the vertical stress and strain required for breakage, and a linear increase in Diametral strength Ds (Fig. 3a). Based on the Brazilian test model, we set Ds = 10.2, 20.0, 56.2, 93.4, 113.4 to 122.7 MPa (corresponding to Cbond & Tbond from 50 MPa to500 MPa) to evaluate its influence on multi-grain fragmentation.
- **Representative element area**: To assess the effect of system size, we performed simulations with a larger number of grains by increasing the model dimensions. Additional simulations were conducted with 20 grains in a 150 × 150 μm^2^ box and 40 grains in a 400 × 400 μm^2^ box. The grain size distribution remained homogeneous across all experiments.
  **Table 2:** Numerical parameters used for the oedometric model, with the bonded Mohr-Coulomb (BMC) contact law for the “reference case” model

Parameters	Value	Physical parameters	Value
*k*_*n*_	2e15 Pa/m	Grain Young modulus *E*	9.8 GPa
*k*_*t*_	2e15 Pa/m	Grain Young modulus *E*	9.8 GPa
*k*_*bond*_	2e15 Pa/m	Grain Young modulus *E*	9.8 GPa
*T*_*bond*_	300 MPa	Diametral strength *Ds*	93.4 MPa
*C*_*bond*_	300 MPa	Diametral strength *Ds*	93.4 MPa
*C*_*free*_	0 MPa	Diametral strength *Ds*	0 MPa
*T*_*free*_	0 MPa	Diametral strength *Ds*	0 MPa
$\:{\mu\:}_{p}$	0.5	–	–
*gap*_*ini*_	4e-10 m	–	–
ϒ	0.3	–	–

The contact friction coefficient (local value) was set to 0.5. Further details on data repeatability and additional results are provided in the Supporting Information (Figures S3 to S5). For each simulation, the vertical stress, measured as the resistive stress on the top block, was plotted alongside the vertical strain to evaluate the mechanical response of multiple grains compacted. The vertical strain is computed as the vertical displacement divided by the zero-load height of the sample. The evolution of damage was continuously tracked across all configurations, providing insight into how each parameter affects grain breakage.

### Results

#### Grain young modulus influence

**Fig. 6:**
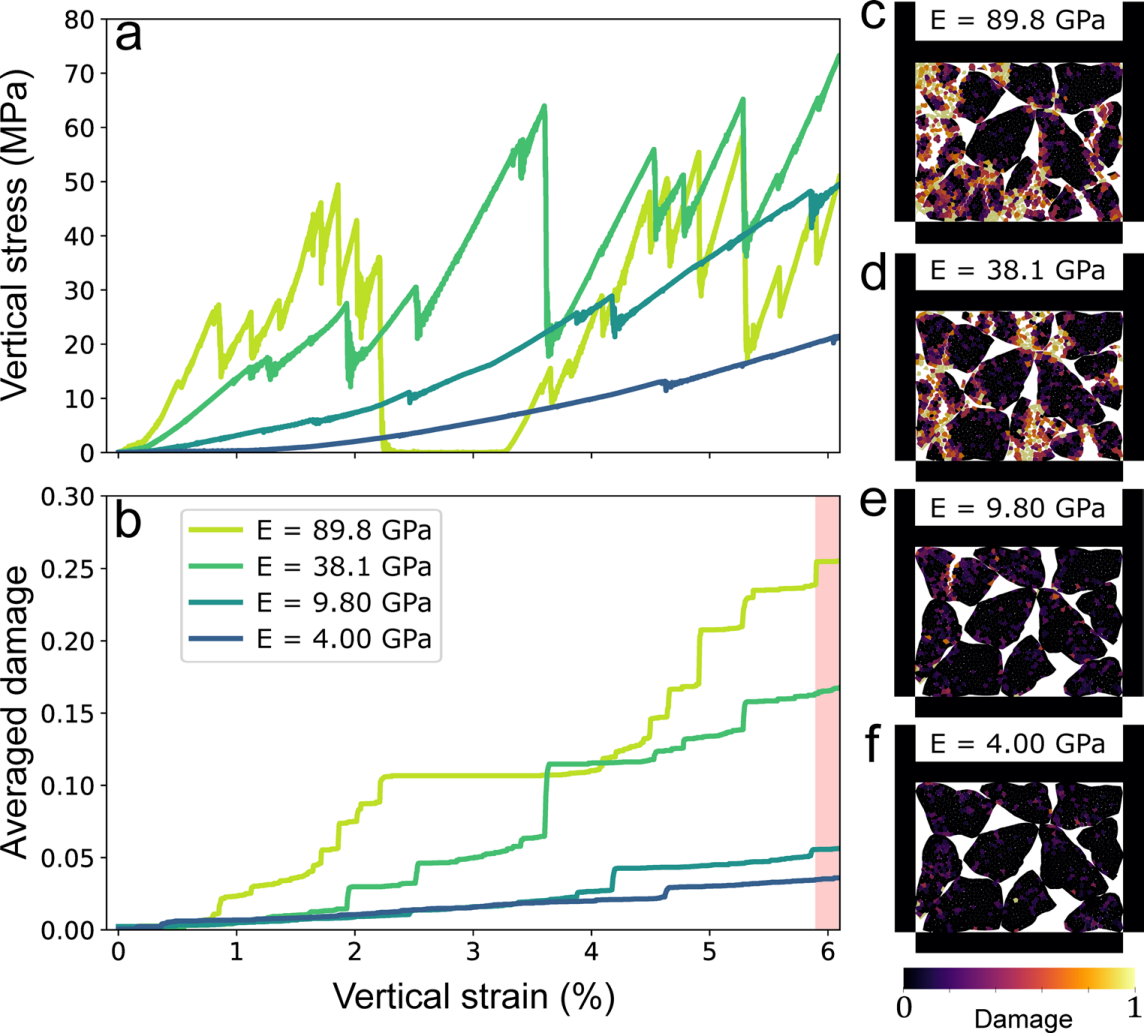
**a** Vertical stress and** b** damage as a function of vertical strain (%), for grain Young modulus values E = 4, 9.8, 38.1, 89.8 GPa. Images** c**–**f** illustrate the damage distribution within the sample at 6% vertical strain. The red area in Figure b indicates the vertical strain corresponding to the extracted microstructures

To assess the role of grain stiffness in multi-grain fragmentation, we tested different Young moduli of grains by varying the normal and tangential contact stiffness values (Sect. 3). Changing the Young modulus gives a different rigidity to the grain, from E = 4 GPa referred to as soft grains, to E = 89.8 GPa, referred to as stiff grains. In multi-grain experiments, grain Young modulus influences both numerical contact overlap (local parameter tested in Sect. 3) and mechanical response, as vertical stress and vertical strain are required for grain breakage. Figure 6 presents the corresponding stress-strain and damage-strain curves.

Vertical stress is increasing with both vertical strain and grain Young modulus (Fig. 6a). Additionally, we can observe that the amount of damage is also increasing with an increase in Young modulus (stiffer grain), from the start of the simulation and toward $\:6\:\%$ of vertical strain (Fig. 6b). Indeed, numerical microstructures at $\:6\:\%$ vertical strain also reveal more pronounced damage for stiffer grains (Figs. 6c–f). A key observation is that increasing grain stiffness leads to a more discontinuous grain breakage process, characterized by multiple small breakage events. This is evident from the stress drops obtained for stiff grains (E = 89.8 GPa), whereas the stress curve for softer grains (E = 4 GPa) remains relatively smooth (Fig. 6a). Furthermore, the increase in vertical stress with vertical strain is not linear, nor is the averaged damage observed within the grains. Very stiff grains can even lead to a complete loss of contact with the loading wall after a large breakage event, leading to sample collapse, with the stress dropping to very low values until the wall catches up and reloads the sample (Fig. 6a, E = 89.8 GPa).

Two distinct regimes can be identified based on grain stiffness. For stiffer grains (E equal to or above 38.1 GPa), the material exhibits marked brittle properties, with important grain fragmentation at 6% of vertical strain. Below this threshold, for similar vertical strain, softer grains did not reach the same vertical stress needed to break grains, indicating a transition in mechanical response (E below 38.1 GPa). Indeed, by being softer, grains can accommodate more strain by elastic deformation before any stress threshold is reached (grain overlap), which can delay the breaking process, which may be reached for higher vertical strain.

#### Diametral strength influence

**Fig. 7:**
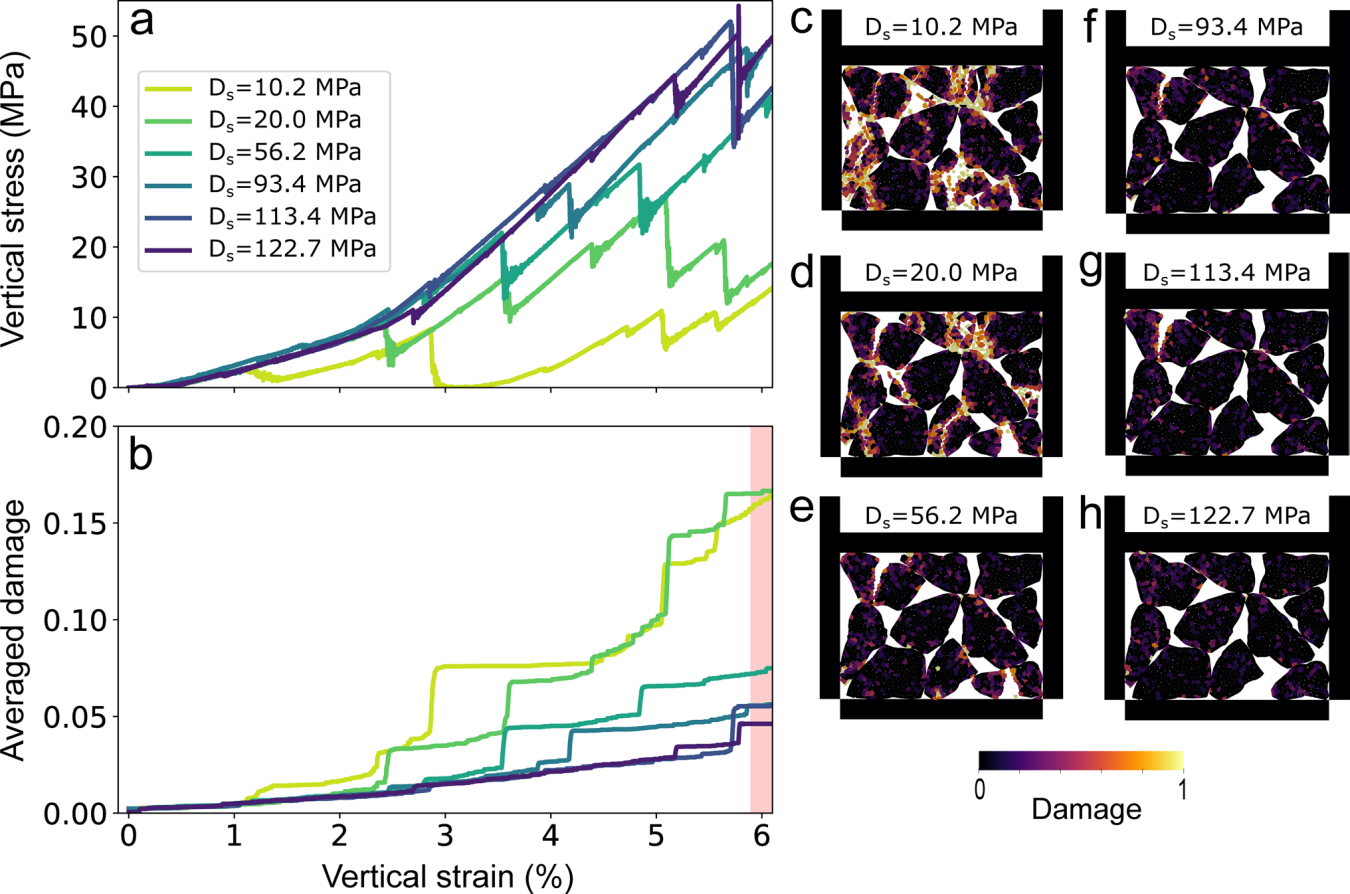
**a** Vertical stress and** b** damage as a function of vertical strain (%), for diametral strength values ($\:Ds$) ranging from 10.22 MPa to 122.74 MPa. Images** c**–**h** illustrate the damage distribution within the sample at 6% vertical strain. The red area in Figure b indicates the vertical strain corresponding to the extracted microstructures

In this section, with multiple grain breaking, diametral strength Ds is varied between 10.22 MPa, referred to as weak grains, to 122.74 MPa, referred to as strong grains. Figure 7 presents the corresponding stress-strain deformation and damage-strain curves.

During the initial loading phase, up to about 1% vertical strain, all simulations exhibit similar vertical stress responses (Fig. 7a). Beyond this point, the mechanical behavior diverges based on grain diametral strength (Ds). Simulations with weaker grains (Ds = 10.22 and 20.01 MPa) show lower vertical stresses accompanied by pronounced fluctuations, driven by frequent grain breakage (Figs. 7a). In contrast, stronger grains (Ds ≥ 93.42 MPa) sustain higher vertical stresses with more stable responses and fewer stress drops (Fig. 7a).

Damage evolution further reflects this distinction: weaker grains accumulate the most damage, as shown in both damage-strain plots (Fig. 7b) and microstructural evidence of intense fragmentation (Fig. 7c and d). For stronger grains, the damage increases more gradually and remains significantly lower, roughly one-third of the damage observed in weak grains at 6% vertical strain (Figs. 7f–h). This suggests that above a certain Diametral strength, the high strength of the grains delays the fragmentation process, which prevents observing it in this range of vertical strain.

Our results indicate that higher Diametral strength (i.e., internal cohesion within subgrains) increases resistance to breakage, particularly beyond 93.42 MPa, where grains exhibit significant strength. To ensure data repeatability and understand if the initial grain arrangement (i.e., coordination number, grain shape) affects the stress-strain curves, we have performed a series of additional simulations that are reported in Figure S3 (Supporting Information), yielding the same behavior.

#### Reference elementary area

To evaluate the influence of system size on the stress-strain behavior, additional simulations were performed using larger numerical samples containing an increased number of grains. We ran one experiment with 20 grains (20G) and another with 40 grains (40G), corresponding to sample sizes that are ~ 1.5 and ~ 2 times larger, respectively, than the reference model. All other simulation parameters were kept consistent with the reference case (see Table 2).

**Fig. 8:**
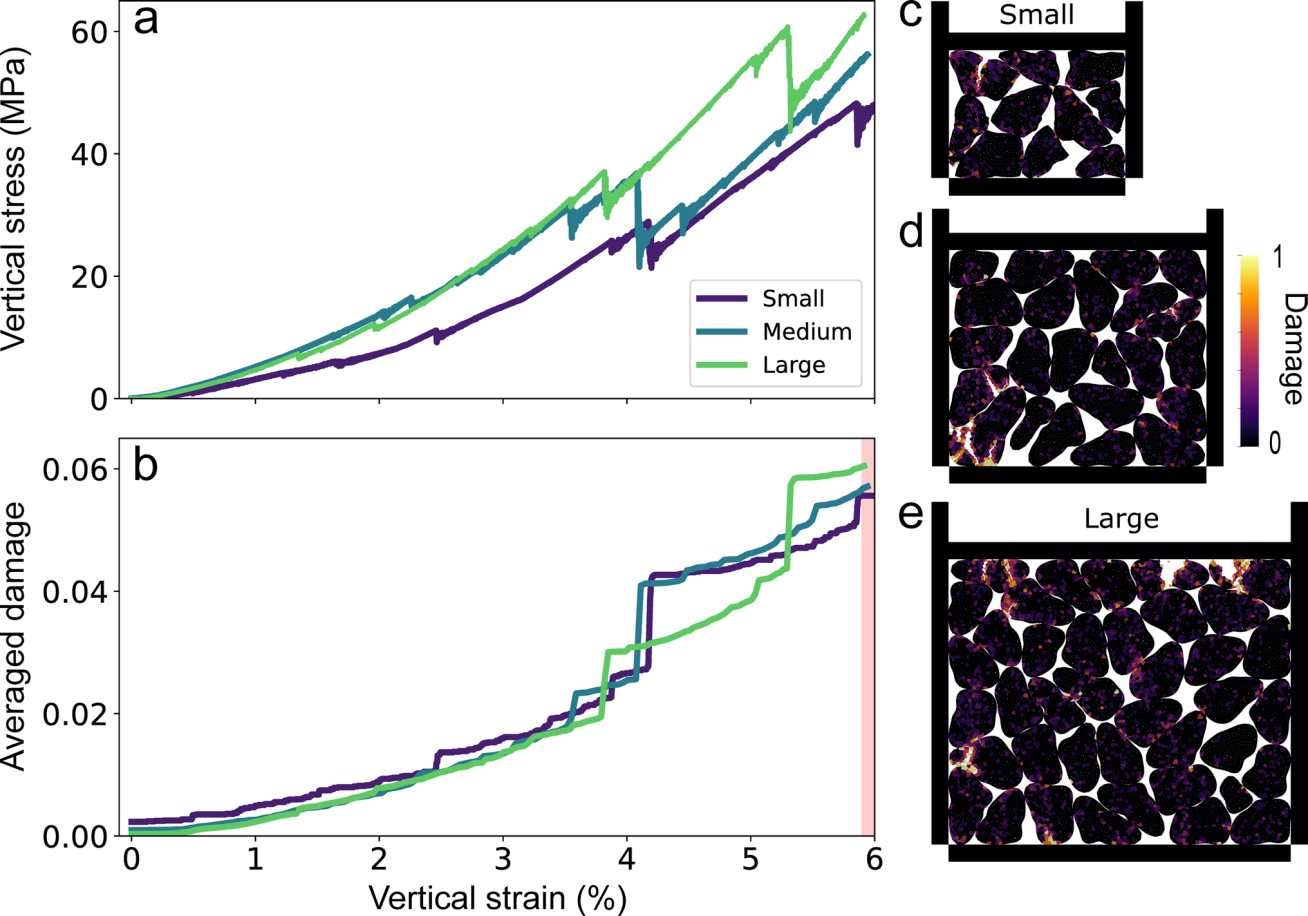
**a** Vertical stress and** b** damage as a function of vertical strain (%) for three different model sizes: 100 × 100 μm^2^ (small) with 10 grains, 150 × 150 μm^2^ (medium) with 20 grains, and 200 × 200 μm^2^ (large) with 40 grains.** c**–**e** Damage distribution within the sample at 5.9% vertical strain. The red area in Figure b indicates the vertical strain corresponding to the extracted microstructures

As shown in Fig. 8a, the evolution of vertical stress exhibits a similar trend across all model sizes. However, the larger samples reach slightly higher vertical stress values at 5.9% vertical strain—60 MPa compared to 45 MPa for the smallest model. This suggests that increasing the model size, and thereby the number of grains, leads to a modest increase in the vertical stress required to achieve a similar level of vertical strain. The observed increase in stress in the larger models may be attributed to a higher coordination number. While the coordination number is not expected to vary with sample size in the bulk limit, it might be affected in the proximity of the walls. The observed stress increase may result from boundary effects and wall proximity, which are more pronounced in smaller samples.

Figure 8b shows that the average damage levels are generally consistent across all simulations at a given vertical strain, which aligns well with the corresponding numerical microstructures (Figs. 8c–e). The average damage increases nearly linearly up to about 4% vertical strain. However, between 4% and 6%, noticeable differences emerge: the timing of sharp damage increases varies slightly between simulations. These abrupt damage jumps also correspond to drops in vertical stress observed in Fig. 8a, indicating episodic grain breakage events during compression. All three models ultimately reach a similar average damage level of approximately 0.055.

It is also worth noting that the relatively small sample sizes used in these oedometric tests were chosen to allow efficient exploration of contact law parameters, rather than to achieve quantitative agreement with laboratory data. Despite the small number of grains, the smallest model consistently captures the mechanical trends of interest.

### Discussion

#### Choice of physical parameters

**Fig. 9:**
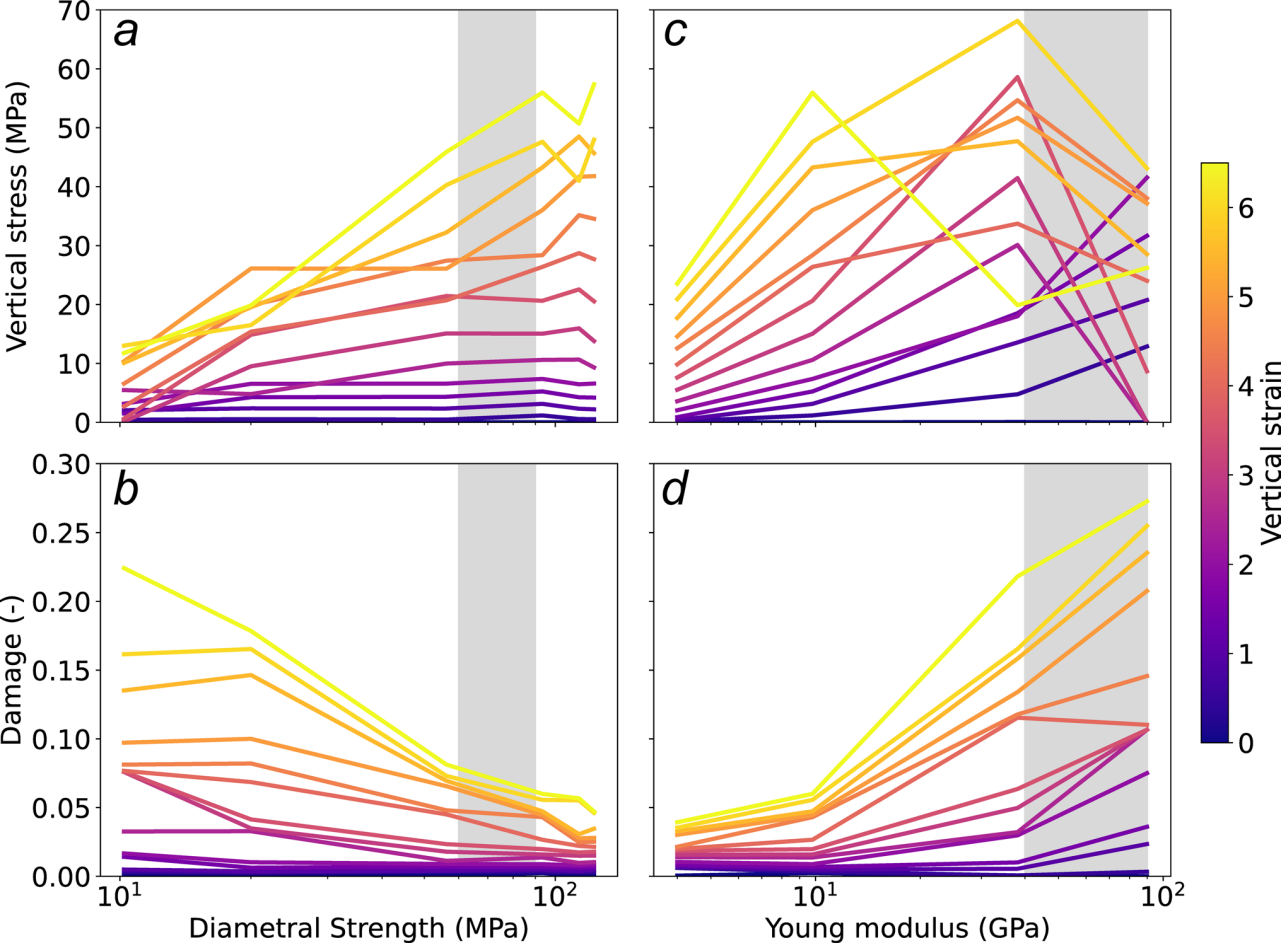
For different strain values:** a** Vertical stress as a function of Diametral Strength Ds (MPa).** b** Damage evolution as a function of Ds.** c** Vertical stress as a function of the grain Young Modulus E (GPa).** d** Damage evolution as a function of E. The colorbar indicates the different strain values from 0 to 6% strain deformation. The grey regions correspond to the selected range of parameters for the next fault model

The primary goal of this oedometric model was to identify physically consistent parameters that can be used to simulate multiple grain breakage within fault gouge experiments. From our numerical experiments, we can examine the evolution of damage and vertical stress as a function of Diametral strength (internal cohesion) and grain Young modulus (contact stiffness) at various strain levels to determine an appropriate parameter range (Fig. 9).

Figure 9a shows that vertical stress stabilizes when the diametral strength Ds ranges between 60–90 MPa. This stabilization suggests that, within this interval, fragmentation progresses more gradually and steadily, unlike at lower Ds values (≤ 60 MPa), where damage is more pronounced (Fig. 9b). At the highest observed vertical strains, the resulting vertical stress falls within the 40–60 MPa range, aligning well with the normal stress intended for the fault gouge model. Additionally, this Ds range corresponds to a continuous increase in damage with increasing strain, as illustrated in Fig. 9b. Although the ratio between tensile and shear strength could play a significant role under shear loading, particularly if the diametral strength varies along the shear direction, we chose not to explore this aspect in the current study. Since the next section serves primarily as a proof of concept for simulating fault gouge shearing with fragmentation, we have adopted a simplified and isotropic strength criterion. Investigating the role of strength anisotropy remains a promising direction for future work. Based on these observations, the Ds range of 60–90 MPa will be adopted for the fault gouge simulations presented in Sect. 5.

Figure 9c and d show how vertical stress and damage evolve with varying grain Young’s modulus E. To realistically model quartz-rich fault gouge, E should lie between 40 and 90 GPa, which corresponds to typical values for quartz [53, 54]. Within this range, damage increases steadily, from 0 to nearly 0.3, as vertical strain progresses from 0% to 6%. Notably, vertical stress initially rises with strain up to around 3%, but exhibits sudden drops when major force chains fail, followed by recovery as strain continues. Within the 40–90 GPa window, the simulations maintain stable behavior: particle overlap remains physically realistic (as seen in Figs. 6, 7 and 8), and damage evolves consistently. Moreover, damage trends in this range are relatively insensitive to minor changes in E, as confirmed by the smooth progression in Fig. 9d. This suggests that the chosen modulus range offers a good compromise between physical realism and numerical stability (see repeatability data in Supporting Information S5). Based on these observations, the E range of 40–90 GPa will be adopted for the fault gouge simulations presented in Sect. 5.

#### Single-grain vs. multiple-grain fragmentation

For a single circular grain (Brazilian test), we observed a linear increase in vertical stress with increasing Diametral strength (Sect. 3.2). However, as we transition to a granular layer composed of multiple angular grains, this relationship becomes less linear due to the added complexity introduced by grain angularity and interactions between particles. The influence of Young modulus in vertical stress and damage also appears less straightforward, as it is strongly influenced by the edges of angular grains used in multiple grain fragmentation and their initial spatial arrangement (Fig. 6).

Given the relatively small size of the model, the breakage of an individual grain is distinctly reflected in the stress and damage evolution (Sect. 3). However, as the model size increases and more grains are introduced, the evolution of both stress and damage becomes more homogeneous. This suggests that a larger number of interacting grains helps distribute stress more evenly throughout the granular layer, reducing the prominence of individual grain breakage events in the overall response (see Sect. 3 and Figs. 6, 7 and 8).

#### Stress drop and elastic energy

The sudden stress drops observed when changing Young’s Modulus are linked to large breakage events that break the load-bearing structure of the material (Fig. 6a). Physically, such events resemble the abrupt failure of force chains, where a rapid release of stored elastic energy leads to a transient loss of stress. In our model, this elastic energy is not confined to discrete contact points but is distributed across the entire surface of the subgrain through the elastic interactions between subgrains. This differs from classical DEM approaches, where energy is primarily stored at contact points. The dynamic effects associated with these failures are especially observed in systems with high contact stiffness, where rupture events occur more abruptly within the simulated deformation window (Fig. 6a). Interestingly, although stiffer contacts (e.g., higher Young modulus) seem to release energy more violently, softer contacts (e.g., softer Young modulus) are capable of storing more energy due to their greater deformation capacity. However, in our simulations, soft grains tend to delay rupture to the point that breakage does not occur within the strain range explored (Fig. 6f). As a result, the observed stress drops are often more intense and less frequent in intermediate stiffness cases (e.g., *E* = 38.1 GPa) than in the stiffest ones (e.g., *E* = 89.8 GPa). This behavior highlights the complex interplay between contact stiffness, energy storage, and failure dynamics in brittle granular systems.

## Simulation of a laboratory fault gouge

### Numerical setup for a fault gouge model

The third type of 2D simulation in this study replicates laboratory Direct Shear (DS) tests, widely used to investigate the role of granular layers (e.g. fault gouge) in frictional sliding of faults [5, 55]. This DS test aims to examine grain fragmentation during fault gouge deformation and its link with frictional behavior, assessing whether our fragmentation model has the potential to reproduce laboratory observations.

**Fig. 10:**
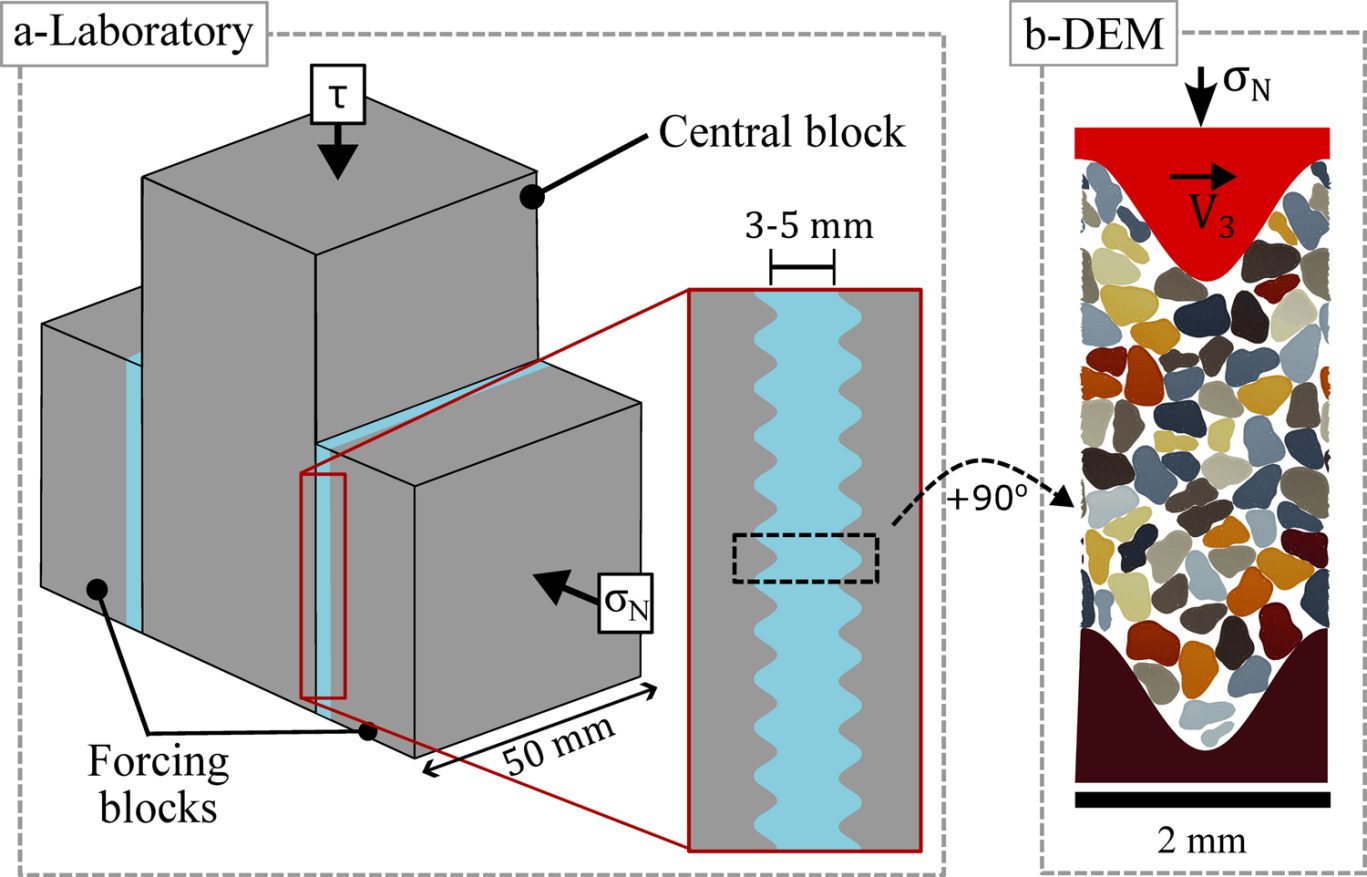
**a** Sketch of the Double Direct Shear (DDS) sample assembly used in a bi-axial apparatus ‘BRAVA2’, with the sample placed in the vessel chamber [44, 55]. Inset, zoom in on the fault gouge layer, with the tooth, in order to avoid any slip at the boundaries.** b** The numerical model is a zoom model of the laboratory experiments

Our numerical setup represents a zoomed-in portion of the laboratory fault gouge (Fig. 10a), measuring 2 × 3 mm^2^ (Fig. 10b). Our model simulates a quartz-rich gouge composed of 70 large angular grains (D50 ≈ 285 μm), further subdivided into 7079 irregular polygonal subgrains with an average diameter of 28.5 μm. The numerical gouge layer is 3 mm thick and confined between two forcing blocks, each 2 mm long. Subgrains are modeled as rigid bodies with a density of 2700 kg/m^3^, and gravity is neglected due to its minimal influence in this context. As in previous models, grain interactions are governed by the BMC contact law, with parameters calibrated from previous simulations (Sects. 3 and 4) and detailed in Table 3.

To ensure shear localization within the gouge and prevent boundary effects, the forcing blocks are equipped with teeth (1 mm in height, spaced 2 mm apart), similar to what is used in the laboratory (Fig. 10a) [44, 55]. The tooth height corresponds to approximately 3.5 times the median grain diameter (D50) and 35 times the subgrain size. Periodic boundary conditions were applied along the lateral edges (green sides, Fig. 2c) to ensure displacement continuity across the shear zone.

To replicate the laboratory procedure, we first allowed the sample to reach steady-state compaction before initiating shear. The lower forcing block is fixed, while a normal stress of σ_N_ = 40 MPa is gradually applied via the vertical loading block, enabling the gouge to compact and stabilize. The sample is then held under this constant load until it attains a steady-state compaction condition. The gouge is then sheared at a constant velocity *V*_3_ = 1 m/s until a total displacement of 2.5 mm is reached. This velocity ensures that the inertial number remains below 10^−3^, keeping the system within the quasi-static granular flow regime and preventing inertial effects [56]. Unlike laboratory experiments that are usually performed at velocities of tens of μm/s, this higher velocity reduces simulation runtime without altering the fault deformation behavior [25].

Throughout the simulation, fault gouge evolution is monitored, tracking mechanical data evolution, such as friction and compaction, and grain breakage under shearing conditions. The macroscopic friction ($\:\mu\:$) (different from the local friction imposed in the model) is calculated as the ratio of shear stress ($\:\tau\:$) to normal stress ($\:{\sigma\:}_{N}$) applied to the fault ($\:\mu\:\:=\:\tau\:/{\sigma\:}_{N}$). Compaction, representing vertical strain, is determined as the relative vertical displacement divided by the initial layer thickness. The evolution of damage (grain fragmentation) is continuously tracked during gouge shearing.

**Table 3:** Numerical parameters used for the DS model, with the bonded Mohr-Coulomb (BMC) contact law. The parameters below are those of the “reference case”

Parameters	Value	Physical parameters	Value
*k*_*n*_	1.3^e^15 Pa/m	Grain Young modulus *E*	88-91 GPa
*k*_*t*_	1.3^e^15 Pa/m	Grain Young modulus *E*	88-91 GPa
*k*_*bond*_	1.3^e^15 Pa/m	Grain Young modulus *E*	88-91 GPa
*T*_*bond*_	300 MPa	Diametral strength *Ds*	–
*C*_*bond*_	300 MPa	Diametral strength *Ds*	–
*C*_*free*_	0 MPa	Diametral strength *Ds*	0 MPa
*T*_*free*_	0 MPa	Diametral strength *Ds*	0 MPa
$\:{\mu\:}_{p}$	0.5	–	–
*gap*_*ini*_	6e-9 m	–	–
ϒ	0.3	–	–

### Results

**Fig. 11:**
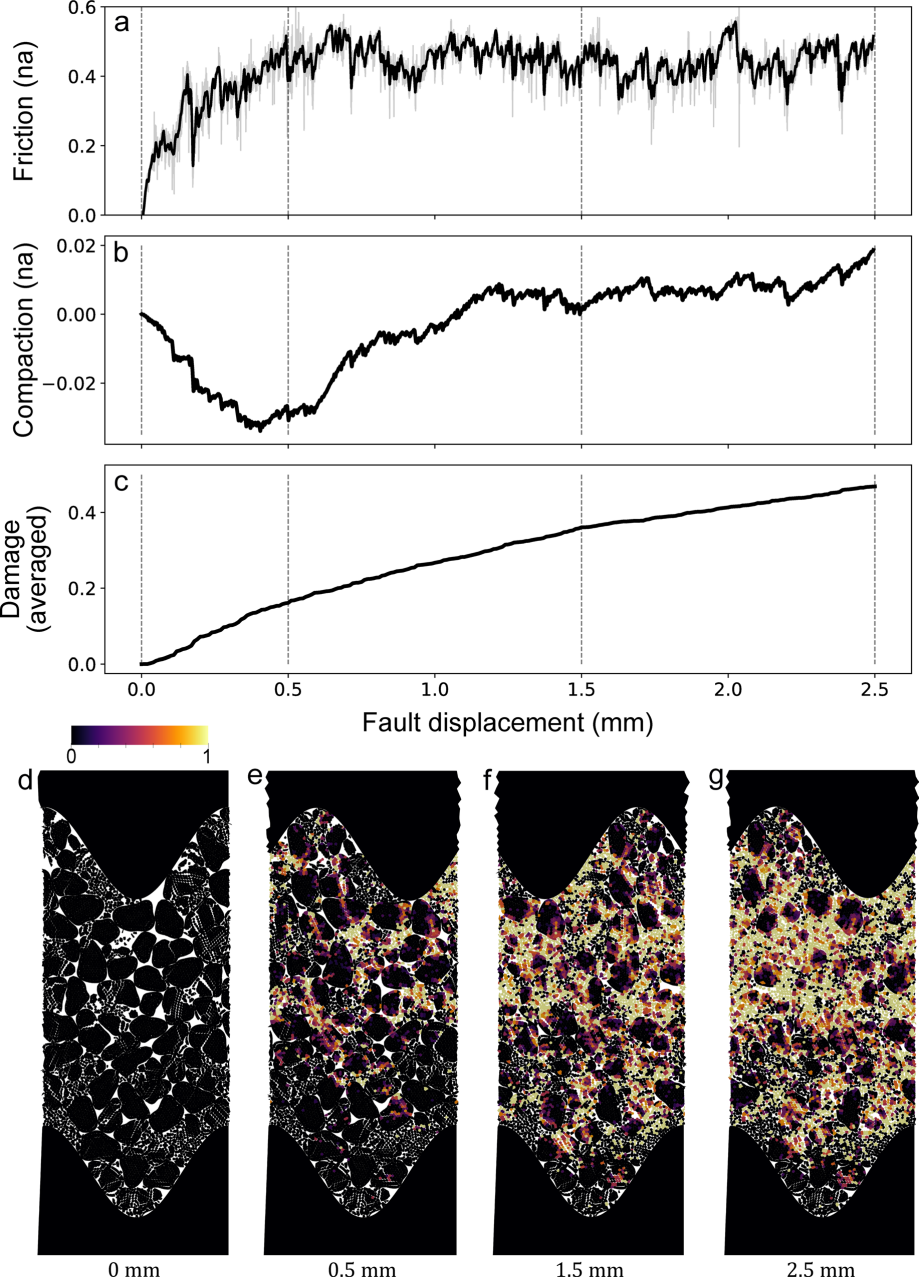
**a** Friction** b**, Compaction, and** c **averaged damage as a function of the fault displacement. Visual outputs of damage during shearing,** d** start of shearing, with initial reset of the relative damage, in order to see the damage only created by shearing,** e** state of the gouge after 0.5 mm shearing,** f** state of the gouge after 1.5 mm shearing, and** g** state of the gouge after 2.5 mm shearing

**Fig. 12:**
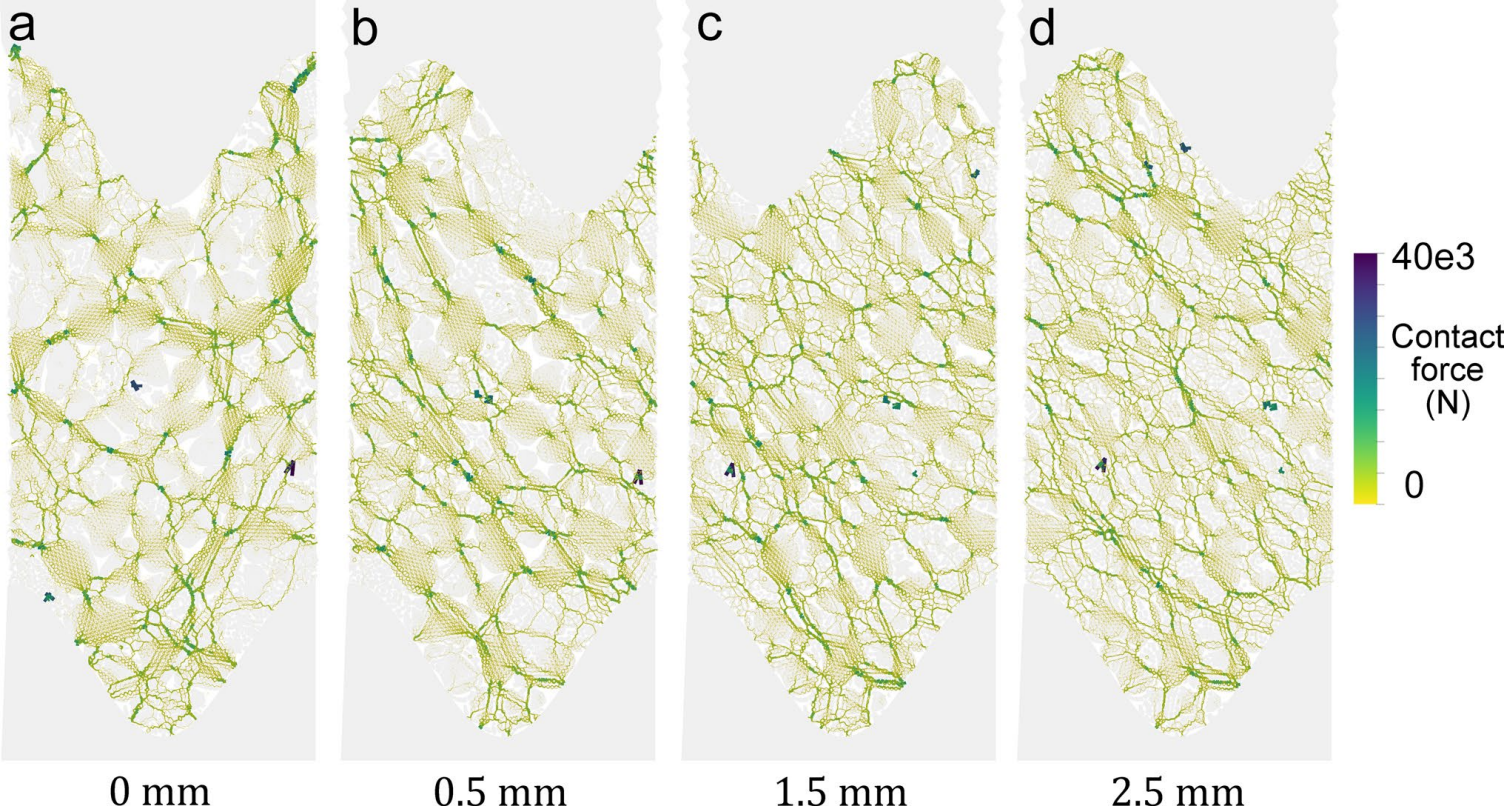
Force chains evolution through fault displacement** a **start shearing,** b** displacement of 0.5 mm,** c** displacement of 1.5 mm, and** d** displacement of 2.5 mm. Force chains’ contact force magnitude ranges from 0 to 40 kN

The fault gouge (represented as granular particles) is first compacted under a normal stress of 40 MPa until steady-state compaction is achieved (Sect. 5.1). Significant damage has already occurred after the gradual application of the normal stress (see Supporting Information S6). Shearing is then driven by the upper wall. Figure 11 illustrates the evolution of friction, compaction, and damage as functions of fault displacement, and Fig. 12 shows the force chains within the gouge with shearing. The analysis identified three distinct frictional stages:

- During the initial stages of shearing, from 0 to 0.5 mm of displacement, friction increases, and substantial compaction occurs, approaching a peak value (Fig. 11a, b). Compaction is known to reflect fragmentation processes due to particle size reduction and grain rearrangement [10, 33, 44]. Indeed, damage increases significantly during the initial micrometers of shearing, accounting for one-fourth of the total damage (Fig. 11c and e). During this stage, the force network transitions from a highly heterogeneous distribution, with isolated groups of grains experiencing minimal load (Fig. 12a), to the formation of force chains oriented at approximately 45 degrees to the shear direction, though still exhibiting some heterogeneity (Fig. 12b).
- From 0.5 to 1.5 mm of displacement, friction reaches a peak value ($\:\mu\:\:=\:0.55$) before gradually transitioning toward a steady state. Compaction reduces (reduced vertical strain), while damage continues to increase as grain breakage remains active throughout the gouge layer (Fig. 11c, f). During this stage, force chains maintain their 45-degree orientation relative to the shear direction but become more uniformly distributed as the system approaches steady-state (Fig. 12c).
- Beyond 1.5 mm of displacement, the system reaches a steady-state friction coefficient of approximately 0.45 (Fig. 11a). Compaction evolution stabilizes (Fig. 11b), and slip becomes localized within a boundary shear band in the upper part of the gouge layer (Fig. 11g). Despite this localization, damage continues to accumulate, indicating that grain breakage persists within the central deformation zone (Fig. 11c). This additional fragmentation occurring within the Central Deformation Zone (increase in damage) highlights the ongoing redistribution and failure of grains under shear stress. Force chains continue to evolve with shearing, highlighting highly stressed zones and suggesting that slip may not yet be fully localized (Fig. 12d).

## Comparison with a laboratory fault experiment

Discrete Element Modeling (DEM) has become essential for understanding fault mechanics, particularly in simulating frictional and contact properties within fault cores [16, 18, 24]. However, many DEM models rely on circular or spherical particles that oversimplify the irregular, angular nature of real fault gouges [4, 57]. This can lead to unrealistic contact mechanics, fracture patterns, and porosity evolution [36, 37, 39]. To address these limitations, we improved a DEM framework featuring angular, breakable grains based on bonded Voronoi tessellations and an inverse Monte Carlo method [40]. Thanks to the same method, grains are further discretized into polygonal subgrains, enabling more realistic fracture propagation and force chains. This approach offers an important step forward in simulating the micromechanics of fragmentation and deformation in fault gouges.

**Fig. 13:**
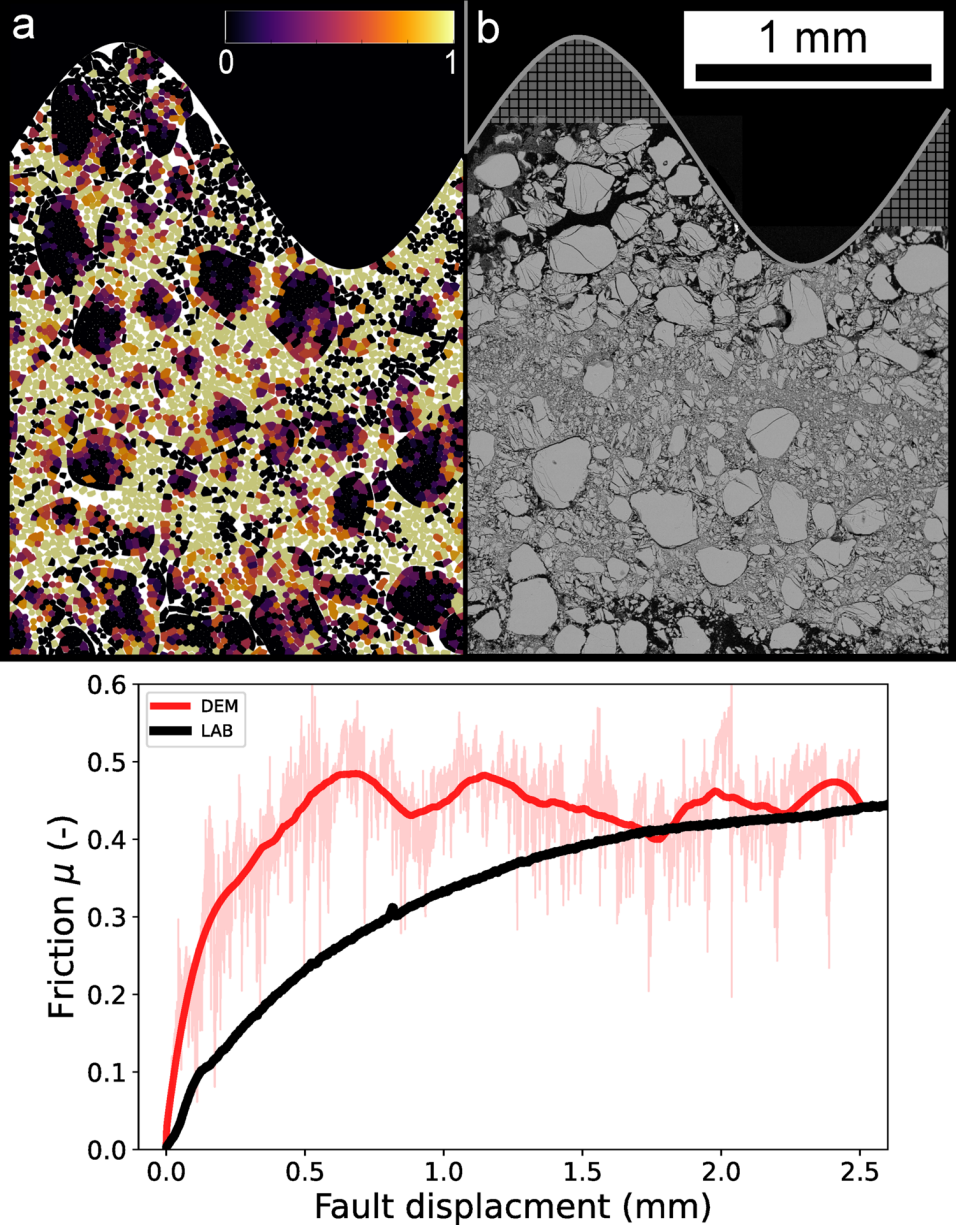
Microstructures at the same scale** a** numerical, after 2.5 mm shearing,** b** SEM backscattered image from a laboratory experiment after 5 mm shearing (F70 grains, initial average grain size equal to 205 μm, double direct shear experiment, shearing velocity 10 μm/s, initial gouge thickness of 5 mm).** c** Friction for the two experiments as a function of the fault displacement (mm) for both laboratory (black curve) and numerical DEM (red curve) experiments. Friction from the laboratory experiment at larger fault displacements can be observed in Supporting Information S7

To evaluate the realism of our model, we compared our simulations (70 quartz grains, D50 = 285 μm, normal stress = 40 MPa) with a laboratory Double Direct Shear (DDS) experiment using the “BRAVA 2” apparatus at the Earthquake Physics and Rock Mechanics Laboratory (La Sapienza, Rome), following the procedure described in Casas et al. [44]. The apparatus applies normal and shear stresses via two orthogonal servo-controlled rams, with load measurements obtained from regularly calibrated strain-gauge load cells with a resolution of less than $\:1\:kN$. In the (DDS) configuration, two identical squared layers of simulated fault gouge ($\:50\:{\mathrm{mm}}$ wide, $\:5\:{\mathrm{mm}}$ thick) were sandwiched between a central block and two side forcing blocks (Fig. 10a). The laboratory gouge sample consisted of F70 quartz grains with an average grain size of 205 μm, slightly smaller than those used in our numerical model. During the experiment, friction evolution was measured as a function of fault displacement, and the deformed microstructure was retrieved at the end of the experiment. Figure 13 compares our simulated microstructure (Fig. 13a) with the SEM (Scanning Electron Microscope) backscattered image of the quartz gouge sheared to $\:5\:{\mathrm{mm}}$ displacement in the laboratory (Fig. 13b), along with the evolution of macroscopic friction (Fig. 13c).

Despite differences in final displacement ($\:2.5\:{\mathrm{mm}}$ in the simulation vs. $\:5\:{\mathrm{mm}}$ in the laboratory) and sample dimensions, both systems exhibit key similarities: large grains remain in the shear zone, while the upper gouge region shows intense grain size reduction (Figs. 11g and 13a and b). Shear localization has been linked to grain size reduction through fragmentation and cataclasis, contributing to mechanical weakening [7, 58, 59]. Our model also reproduces structural features such as the formation of B-shear zones (Fig. 11g), consistent with laboratory evolution from early-stage Riedel bands to more developed B- and Y-shears as displacement increases [60] (Fig. 13b). Within these B-shears, we observe fragmentation down to the minimum subgrain size (28.5 μm, Yellow color damage, Fig. 13a), along with grain edge chipping and rounding, microstructural features observed in both simulations and backscattered SEM images. The smallest grains observed in laboratory shear zones can approach nanometric scales [61, 62], reducing the porosity within the B-shear (Fig. 13a, b). However, in the model, the imposed subgrain resolution was fixed to balance the resolution with computational efficiency. This simplification means that finer fragmentation is not fully captured, but the key aspects of grain breakage and shear mechanisms are represented.

Friction evolution in our numerical model is of similar magnitude to that observed in laboratory experiments, yet some discrepancies remain, though differences appear in the early stages of the experiments (Fig. 13c). The simulated gouge shows a stiffer response during initial displacement, which may result from differences in initial porosity from over-compaction during sample preparation in MELODY. Differences in initial grain size and layer thickness may also influence compaction and stiffness of the granular layer.

At steady state, our 2D model reaches a friction coefficient of ~ 0.45, slightly lower than typical laboratory values for quartz (~ 0.5–0.6) [44], which achieve steady-state after greater displacement (Supporting Information S7). Although the two-dimensional nature of the model limits out-of-plane grain interactions, which can reduce steady-state friction (force transmission and grain rearrangement) in comparison to its 3D equivalent [63], dimensionality alone might not fully explain the discrepancies. The use of periodic boundary conditions in the model, while necessary to maintain computational feasibility, suppresses boundary-driven heterogeneities and processes such as gouge layer thinning, which are common in laboratory setups. Calibrating the material parameters more carefully could also improve the correspondence between numerical and laboratory data, though this was not the focus of the present study and remains a demanding task. Future improvements could include larger domains (to test the influence of the model size on the timing and development of shear localization), 3D simulations, or more realistic boundary conditions, though at significantly increased computational cost [10, 26, 64].

The framework proposed in this study provides a solid foundation for investigating how initial grain properties, especially size and angularity, affect fragmentation, fault strength, and shear localisation. While previous studies have modeled angularity and grain fragmentation using bonded circular particles (i.e., clumped spheres) [65, 66], our approach introduces a significant advance by generating truly angular grains and a dynamic fragmentation mechanism based on local stress thresholds. These grains are further discretized into polygonal subgrains, enabling more realistic fracture propagation and force transmission than circular particles. This enhanced representation of grain shape and internal structure allows us to capture the spatial evolution of damage and strain localization more realistically. Moreover, the model’s scalability and flexibility under varying stress conditions make it suitable for a wide range of geological and engineering applications.

Finally, this framework opens new avenues for integration with emerging multiscale models, such as heterarchical multiscale modeling [67] and the “Population balance” method [68], which aim to embed fragmentation processes into continuum or hybrid formulations. By bridging microstructural mechanics with macroscopic behavior, our approach lays the groundwork for future studies of fault gouge evolution across scales.

## Conclusion

This study presents an improved DEM-based approach to model grain fragmentation in fault gouge, overcoming the limitations of traditional models that use simplified grain geometries. By incorporating angular, breakable grains, our method captures key micro-mechanical processes that influence fault strength and deformation. Through a sequence of numerical models, including single-grain fragmentation (Brazilian test), multi-grain compression (oedometric model), and shearing fault gouge simulations, we refine numerical parameters and analyze progressive breakage, grain rearrangement, and strain localization.

Unlike conventional DEM approaches with fixed grain sizes or spherical particles, our model allows dynamic grain breakage, leading to continuous evolution in porosity, coordination number, and force chains. This enhances the accuracy of reproducing fault gouge microstructures and mechanical responses observed in laboratory experiments. By resolving discrepancies in previous DEM studies, particularly regarding shear strength evolution, compaction, and localization patterns, our findings emphasize the critical role of fragmentation in controlling the long-term mechanical behavior of fault gouges.

While computational scalability remains a challenge, future work will extend this framework to larger domains and integrate more realistic grain size distributions. Overall, this approach provides a significant advancement in fault mechanics simulations, bridging the gap between numerical modeling and laboratory observations while offering deeper insight into the microphysical processes governing fault slip.

## Supplementary Information

Below is the link to the electronic supplementary material.

Supplementary Material 1

Supplementary Material 2

## Data Availability

All simulations were performed with the open-source software MELODY version 3.95 and 4.00dev (DOI: 10.5281/zenodo.4305614) developed and described in Mollon (2018). The code is available for downloading at https://github.com/GMollon/MELODY2D. The collected data analyzed in this paper are available at [69]. For any further requests, please don’t hesitate to contact the corresponding author at nathalie.casas@uniroma1.it.
