# Supplementary material for: Angular grain fragmentation with DEM modeling: application to fault gouge shearing
Source: Granul Matter. 2025 Oct 21;27(4):109. doi: 10.1007/s10035-025-01578-9 (PMC12540612; doi:10.1007/s10035-025-01578-9)
Supplement: Supplementary file 2 — Supplementary Material 2 [file 10035_2025_1578_MOESM2_ESM.pdf]

Supporting Information for

# Angular grain fragmentation with DEM modeling: application to fault gouge shearing

Nathalie Casas [1*], Guilhem Mollon [2], and Marco Maria Scuderi [1]

[1]Dipartimento di Scienze della Terra, La Sapienza Università di Roma, Rome, Italy
[2]LaMCoS, CNRS UMR5259, INSA Lyon, Lyon, France

**Contents of this file**

Figures S1 to S7

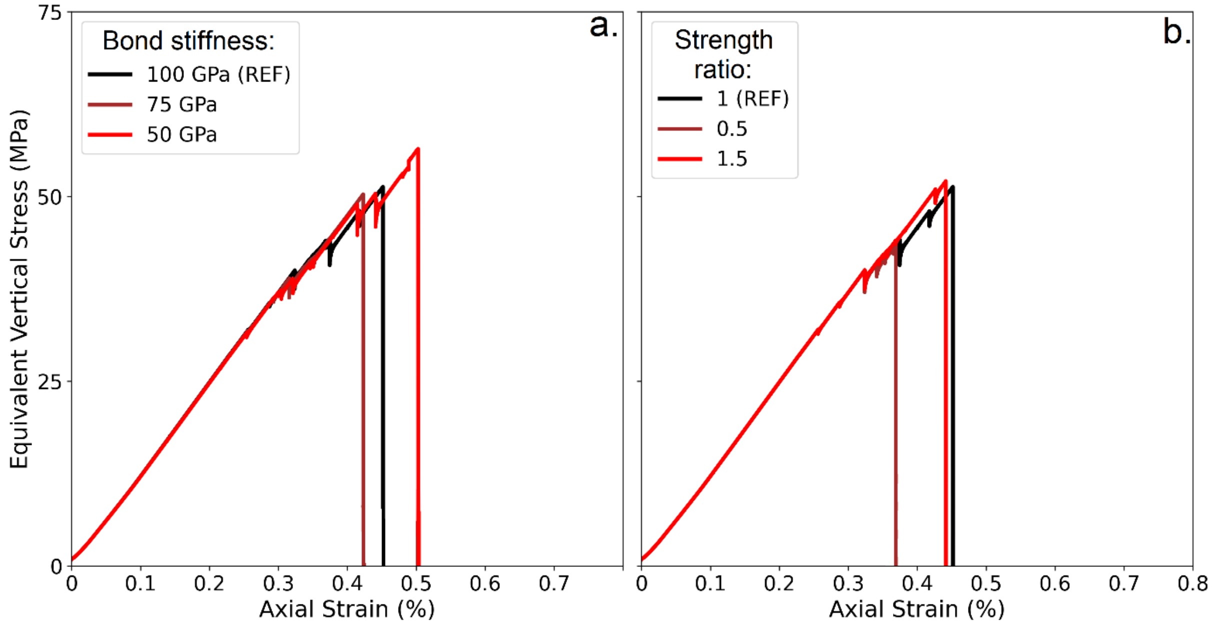


Figure S1. Equivalent vertical stress (MPa) as a function of the vertical strain (%), for (a) different bond stiffnesses $k_{bond}$; (b) tensile ($T_{bond}$) and tangential ($C_{bond}$) cohesive stresses, with varying ratios between the two. Additionally, the bond stiffness $k_{bond}$ (e.g., section 2.2) – which quantifies the stiffness of the cohesive bond itself – was independently varied within the same range as $k_n$ and $k_t$ to assess its role in fragmentation behavior. For the reference model, the stiffnesses ($k_n, k_t, k_{bond}$ ) are set to $5.6e15\ Pa/m$ (Table 1, associated paper). These values were first modified equally and then adjusted with relative ratios of 0.5, 1, and 1.5 to explore their differential effects on grain cohesion.

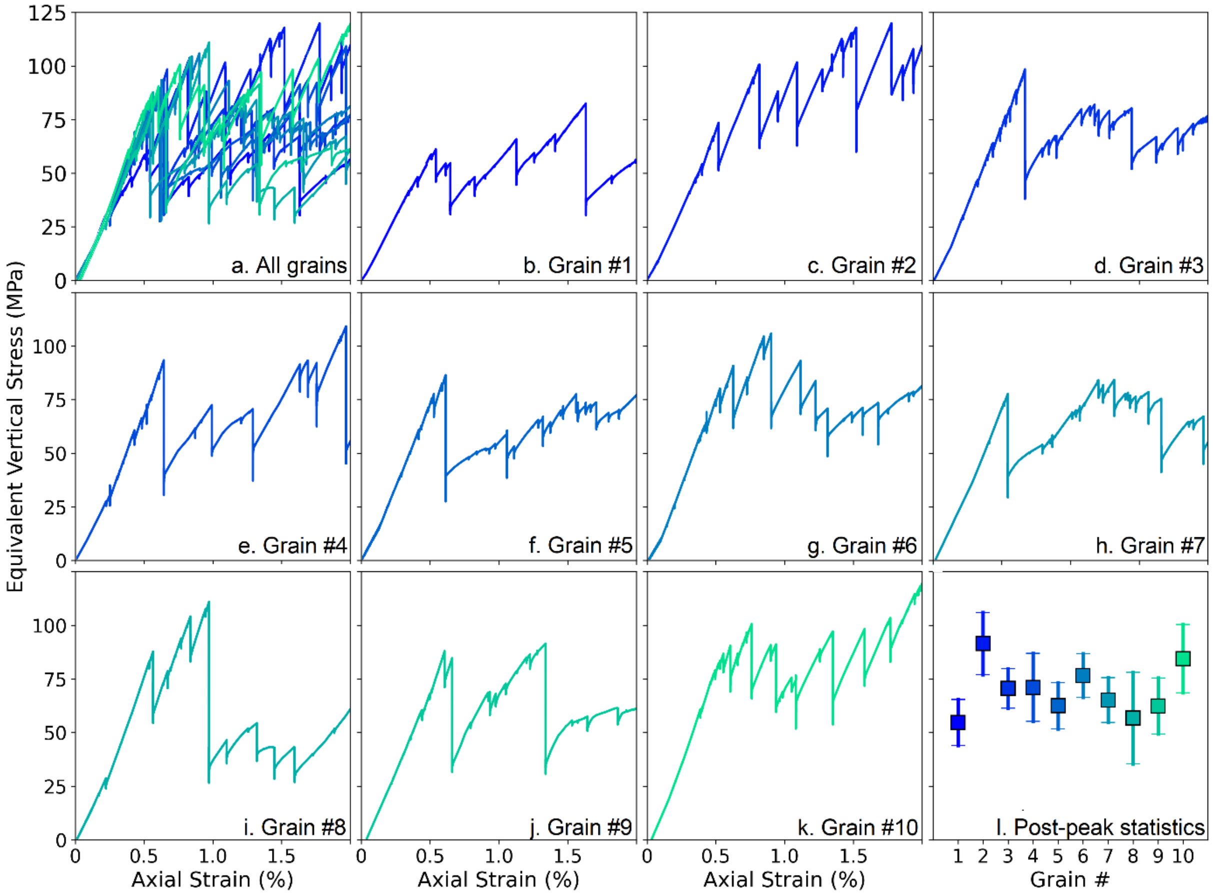


Figure S2. Ten grains were subjected to repeated tests under lateral confinement of 20 MPa. (a)-(k) Equivalent vertical stress (MPa) as a function of axial strain (%) under laterally confined conditions. (l)The final panel shows the average vertical equivalent stress in the post-peak regime (± one standard deviation) for each grain.

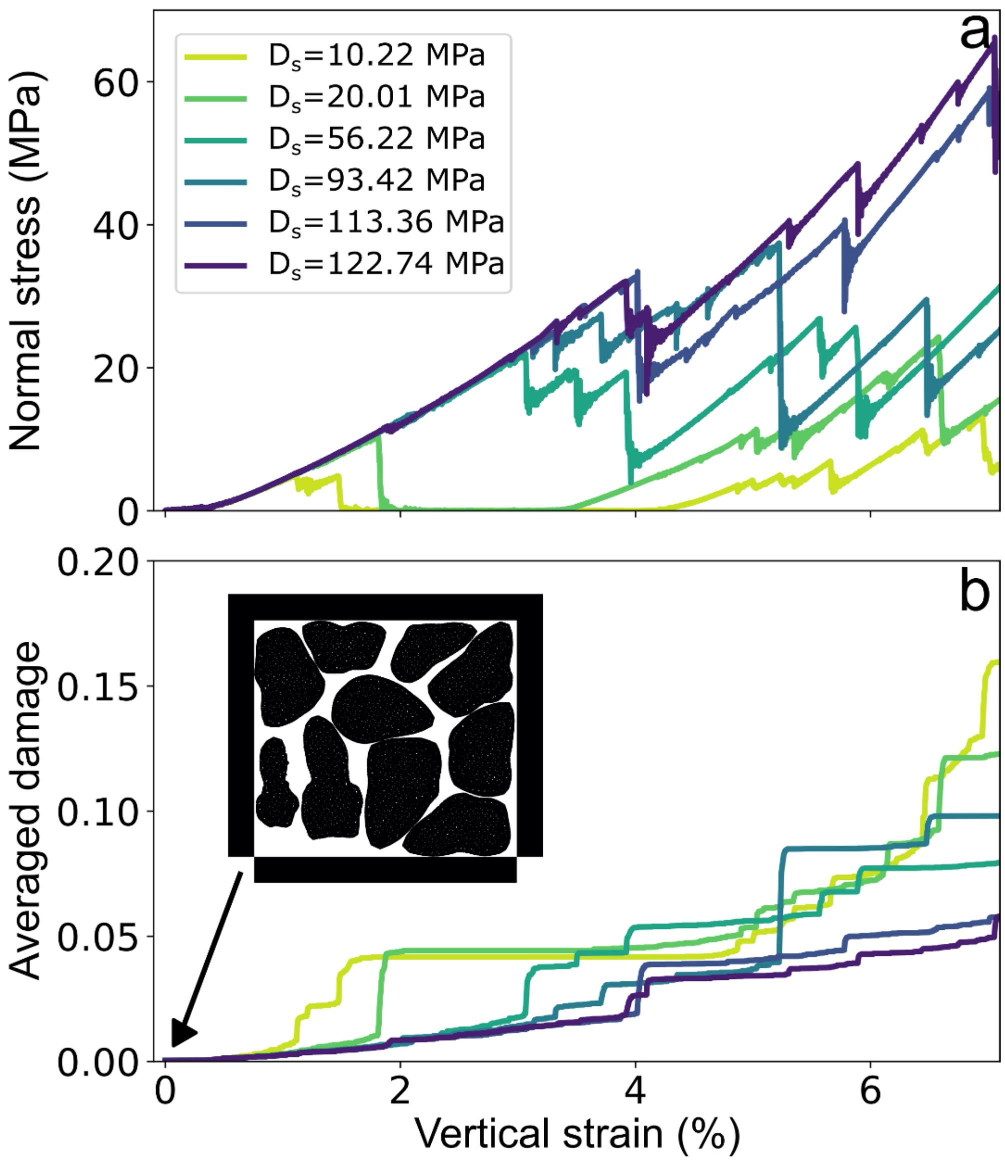


Figure S3. (a) Vertical stress (normal stress) and (b) averaged damage as a function of vertical strain (%), for cohesive stress values $C_{bond} = T_{bond}$ ranging from $50\ MPa$ to $500\ Mpa$, corresponding to Diametral strength value ranging from $10.22\ MPa$ to $122.74\ MPa$ for a different initial grain configuration than the reference case.

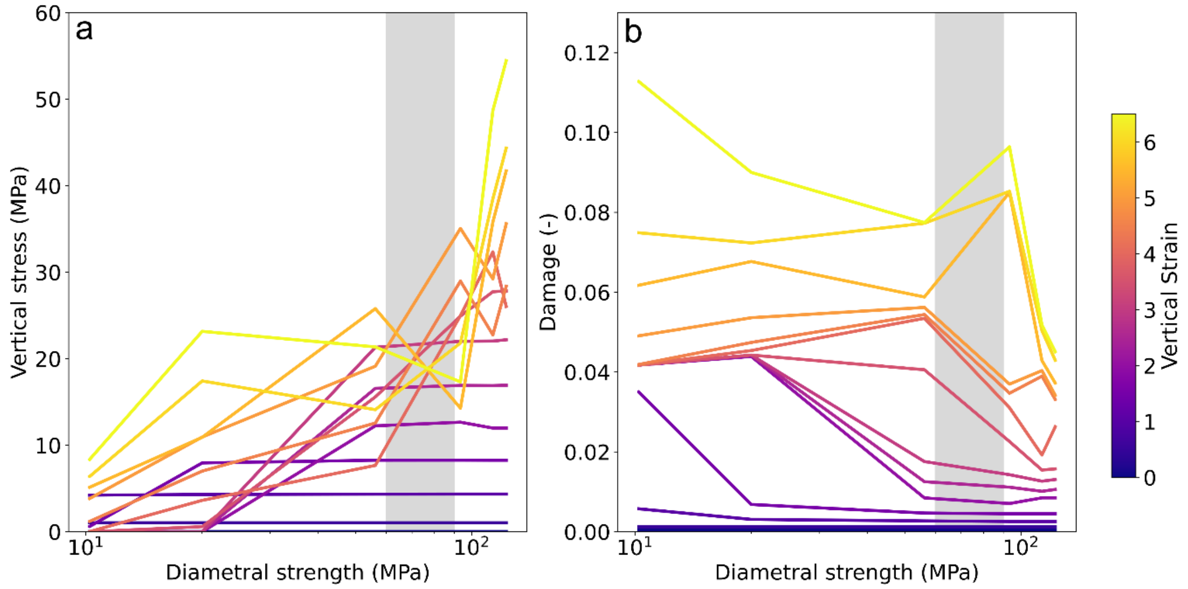


Figure S4. (a) Vertical stress and (b) Averaged damage as a function of the Diametral strength (cohesion stresses) for different strain values, for the second initial grain configuration (Figure S2 above).

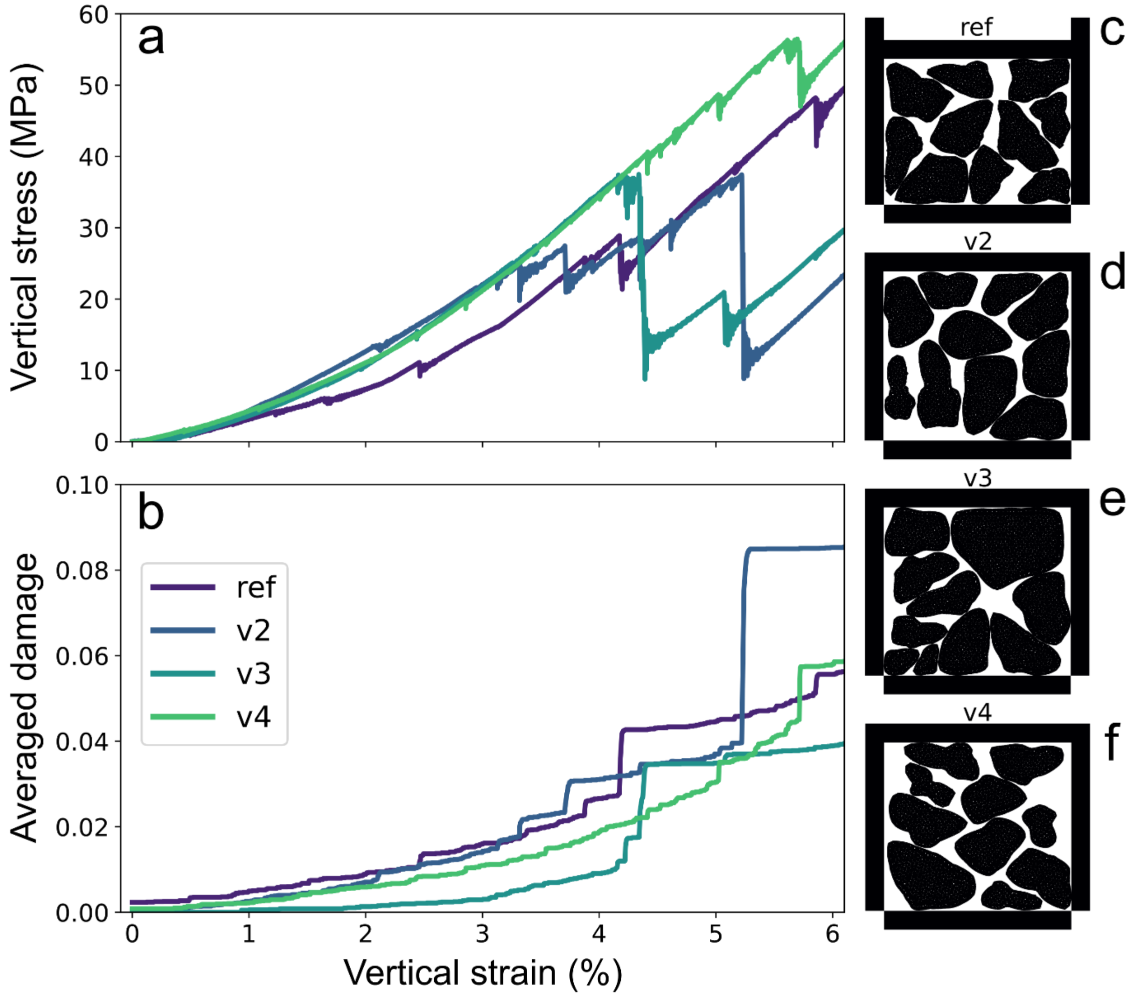


Figure S5. (a) Vertical stress and (b) averaged damage as a function of vertical strain (%), for different initial grain configurations. The numerical parameters used are those of the reference experiment. Images (c to f) illustrate the damage distribution within the sample at 0% vertical strain. The configuration v2 is the one used for figures S2 and S3.

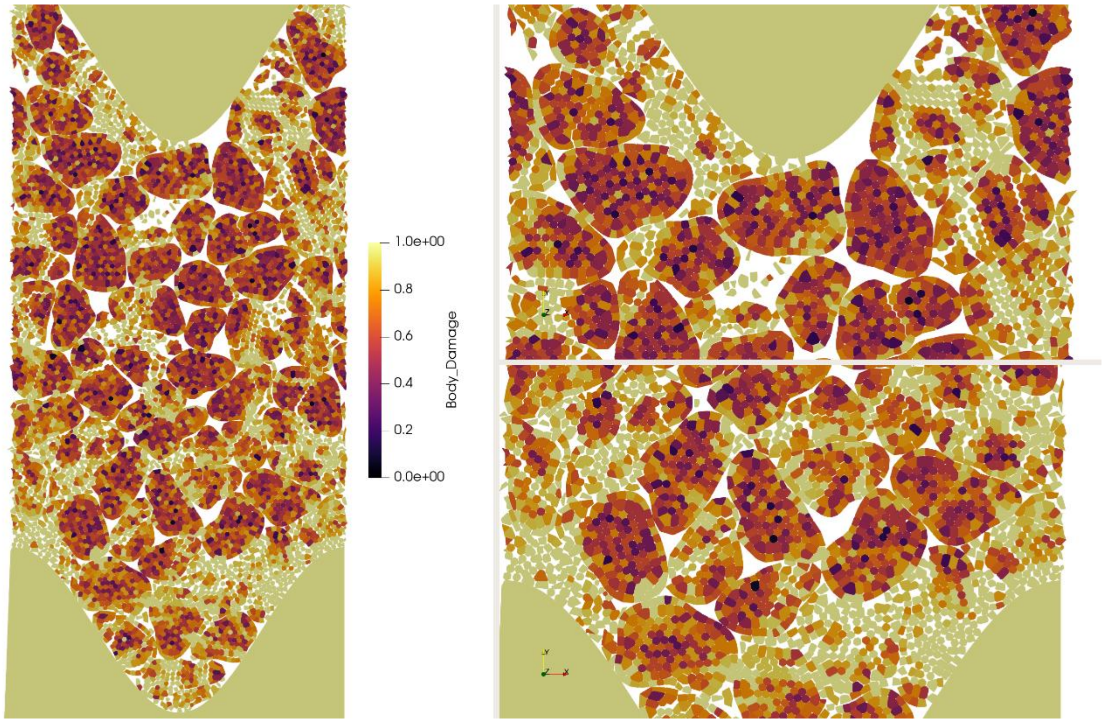


Figure S6. Compaction within the fault gouge just before shearing (end compaction). Yellow grains have a damage of 1 (unbonded subgrains), and black ones are almost not damaged. The top right panel corresponds to a zoom of the top part of the left figure, and the bottom right panel corresponds to a zoom of the bottom part of the left figure.

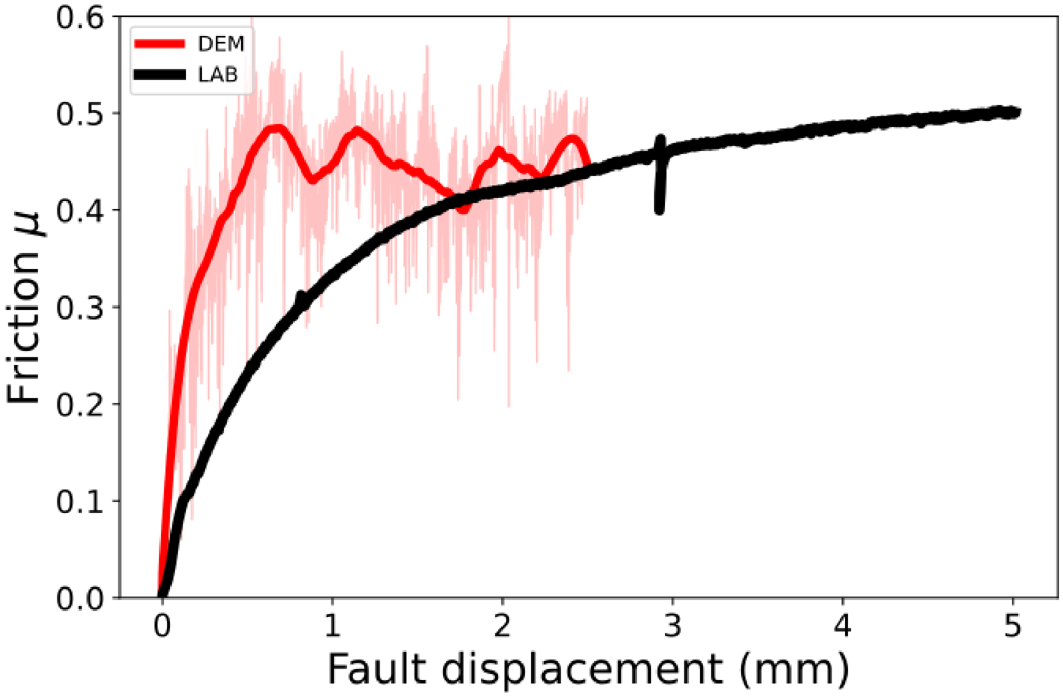


Figure S7. Friction for the two experiments as a function of the fault displacement (mm) for both laboratory (black curve) and numerical DEM (red curve) experiments.
